# *In silico* and *in vitro* studies of the reduction of unsaturated α,β bonds of *trans*-2-hexenedioic acid and 6-amino-*trans*-2-hexenoic acid – Important steps towards biobased production of adipic acid

**DOI:** 10.1371/journal.pone.0193503

**Published:** 2018-02-23

**Authors:** Emma Karlsson, Jae Ho Shin, Gunnar Westman, Leif A. Eriksson, Lisbeth Olsson, Valeria Mapelli

**Affiliations:** 1 Department of Biology and Biological Engineering, Division of Industrial Biotechnology, Chalmers University of Technology, Gothenburg, Sweden; 2 Department of Chemistry and Chemical Engineering, Division of Chemistry and Biochemistry, Gothenburg, Sweden; 3 Department of Chemistry and Molecular Biology, University of Gothenburg, Gothenburg, Sweden; Universidade Nova de Lisboa Instituto de Tecnologia Quimica e Biologica, PORTUGAL

## Abstract

The biobased production of adipic acid, a precursor in the production of nylon, is of great interest in order to replace the current petrochemical production route. Glucose-rich lignocellulosic raw materials have high potential to replace the petrochemical raw material. A number of metabolic pathways have been proposed for the microbial conversion of glucose to adipic acid, but achieved yields and titers remain to be improved before industrial applications are feasible. One proposed pathway starts with lysine, an essential metabolite industrially produced from glucose by microorganisms. However, the drawback of this pathway is that several reactions are involved where there is no known efficient enzyme. By changing the order of the enzymatic reactions, we were able to identify an alternative pathway with one unknown enzyme less compared to the original pathway. One of the reactions lacking known enzymes is the reduction of the unsaturated α,β bond of 6-amino-*trans*-2-hexenoic acid and *trans*-2-hexenedioic acid. To identify the necessary enzymes, we selected *N*-ethylmaleimide reductase from *Escherichia coli* and Old Yellow Enzyme 1 from *Saccharomyces pastorianus*. Despite successful *in silico* docking studies, where both target substrates could fit in the enzyme pockets, and hydrogen bonds with catalytic residues of both enzymes were predicted, no *in vitro* activity was observed. We hypothesize that the lack of activity is due to a difference in electron withdrawing potential between the naturally reduced aldehyde and the carboxylate groups of our target substrates. Suggestions for protein engineering to induce the reactions are discussed, as well as the advantages and disadvantages of the two metabolic pathways from lysine. We have highlighted bottlenecks associated with the lysine pathways, and proposed ways of addressing them.

## Introduction

The biobased production of adipic acid could be used to replace the current petrochemical-based production route and thus contribute to the more sustainable production of this platform chemical, which is used primarily for the production of nylon. Lignocellulosic materials from forestry and agricultural activities are interesting biobased raw materials for the replacement of oil-based raw material. The sugars in lignocellulose biomass, including glucose and other monosaccharides, can be released after pretreatment and hydrolysis, followed by microbial conversion, using metabolically engineered microorganisms, to convert glucose to adipic acid. Adipic acid is not naturally produced microbially to any great extent, and engineering microorganisms with an efficient metabolic pathway for the conversion of glucose into adipic acid presents a considerable challenge. In recent years, several metabolic pathways have been proposed, and some have been demonstrated to be functional [1–6]. However, titers and yields are far from being industrially relevant. The choice of an efficient metabolic pathway together with efficient enzymes for the targeted metabolic pathway are important for improving the titers and yields of adipic acid.

**Fig 1:**
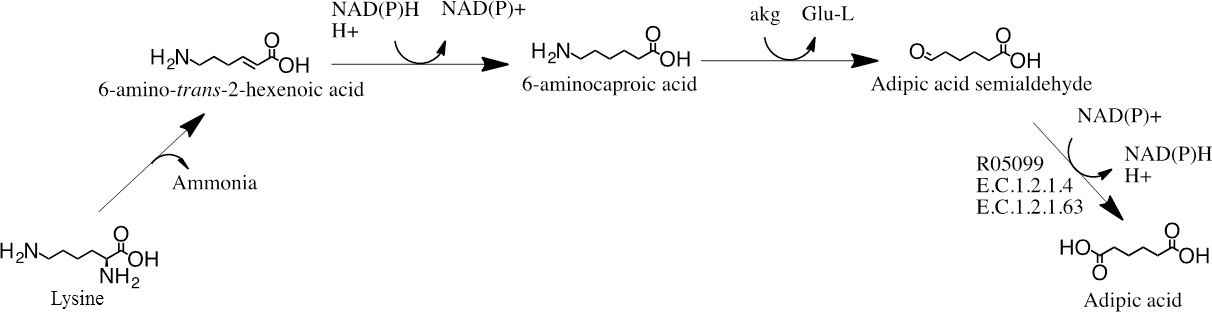
Metabolic pathway for the conversion of lysine to adipic acid proposed by Burgard A. et al. [1]. The reaction number R05099 according to the Kyoto Encyclopedia of Genes and Genomes is given for the known reaction. For known enzymes, the E.C. numbers according to the Braunschweig Enzyme Database are given. akg = alpha-ketoglutaric acid, Glu-L = L-glutamic acid.

One possible pathway, that we have studied, is based on lysine conversion [1] (Fig 1). The theoretical maximum yield to adipic acid from glucose *via* the lysine pathway is reported to be between 40–50% [1]. Lysine is a ubiquitous metabolite that can be naturally synthesized from glucose by most microorganisms and it is also produced industrially by microorganisms at large scale [7,8]. In the first enzymatic reaction step, the NH_2_ group located on the α-carbon is removed from lysine *via* a carbon-nitrogen lyase, forming 6-amino-*trans-*2-hexenoic acid. In the second enzymatic reaction, the unsaturated α,β bond of 6-amino-*trans-*2-hexenoic acid is reduced by an oxidoreductase, utilizing the cofactor NAD(P)H, to form 6-aminocaproic acid. In the third reaction, the amino group of 6-aminocaproic acid is transferred to α-ketoglutaric acid by a transaminase to form adipic acid semialdehyde and L-glutamic acid. In the fourth and final reaction, adipic acid semialdehyde is converted into adipic acid *via* an oxidoreductase utilizing NAD(P)^+^ as cofactor. The main strength of this pathway, compared to many of the other proposed pathways for the production of adipic acid, is that the metabolic pathway from lysine to adipic acid is neutral in terms of redox potential, hence the overall intracellular redox balance is not affected, reducing the risk of lowering the overall yield due to the production of undesirable by-products. While lysine biosynthesis consumes 4 moles of NADPH [9,10], NADPH is not additionally consumed from converting lysine to adipic acid (Fig 1). Synthetic pathways, that are redox neutral, have been successfully introduced into lysine-overproducing strains for direct production of the lysine-derived value-added chemicals from glucose [11–14]. Direct production of 1,5-diaminopentane from glucose using engineered *Corynebacterium glutamicum* with as high as 50% molar yield [15] has been reported, while the maximum theoretical yield for lysine from glucose is reported to be 75% molar yield [16]. Such pathways have also been introduced into microorganisms for conversion of extracellularly supplemented lysine [17,18] with as high as 99.9% molar yield [19]. However, the main challenge in this adipic acid pathway (Fig 1) is that for three of the four metabolic reactions there is no known enzyme that can perform the required reaction efficiently. It is thus necessary to identify efficient enzymes, or to find an alternative pathway with known efficient enzymes.

The primary aim of the present study was to investigate the possibilities and limitations of the lysine pathway bearing in mind that the pathway includes several unknown enzymes. Firstly, efforts were made to identify an alternative pathways from lysine to adipic acid with the aim of reducing the number of unknown enzymes. Efforts were also made to identify enzymes capable of reducing the intermediates with unsaturated α,β bonds in the pathways namely 6-amino-*trans*-2-hexenoic acid (in the pathway shown in Fig 1) and *trans*-2-hexenedioic acid (in the alternative pathway found). Enoate reductases are capable of reducing the unsaturated α,β bonds in a range of substrates [20,21]. However, enoate reductases are sensitive to oxygen, due to the presence of iron-sulfur clusters, and the pathway from lysine requires aeration for maximal yield [1]. Therefore, enoate reductases were not considered in the present study. Instead the enzyme *N*-ethylmaleimide reductase (NemA) from *Escherichia coli* was chosen since it has been reported to convert 6-amino-*trans*-2-hexenoic acid, an intermediate of the original pathway [1], to 6-aminocaproic acid, although with a very low yield and productivity (<0.5% mol product *per* mol substrate after 48 hours of incubation) [22]. While the natural function of NemA is yet to be illustrated, it is known to confer *N*-ethylmaleimide resistance to *E*. *coli* [23]. We also chose the closely related Old Yellow Enzyme 1 (Oye1) from *Saccharomyces pastorianus* [24], which the natural function is also yet to be illustrated despite extensive studies for decades [25]. The enzyme Oye1 is known to reduce unsaturated α,β bonds in a broad range of substrates, including aldehydes and ketones [26], but its potential when acting on carboxylic acids is less promising [27–29]. Therefore, the secondary aim of this study was to carefully investigate the possibilities and limitations of reducing the unsaturated α,β bonds of the carboxylic acid intermediates 6-amino-*trans*-2-hexenoic acid and *trans*-2-hexenedioic acid. A combined approach, in which docking of the enzymes and the substrates *in silico* was combined with experimental work *in vitro* gave us a detailed understanding of the interactions between the enzymes and the substrates, and helped to identify the most important obstacles that must be overcome in order to efficiently reduce the unsaturated α,β bonds of 6-amino-*trans*-2-hexenoic acid and *trans*-2-hexenedioic acid.

## Materials and methods

### Preparation of the ligand for docking

The molecular structures of *trans*-2-hexenal, *trans*-2-hexenoic acid, *trans*-2-hexenedioic acid, and 6-amino-*trans*-2-hexenoic acid, were constructed using MOE (Ver. 2014.09).

### Homology modeling

Homology modeling of the NemA protein (UniProt P77258) was performed using Prime (Ver. 4.1) of Maestro Ver. 10 (Schrödinger LLC). The crystal structure of 3P7Y [30] was used as a template for NemA homology modelling. The identities were 87% and the positives 94%. The NemA model was loaded using MOE for the subsequent preparation step.

### Receptor preparation

Structures of Oye1 (PDB code 1OYB) [25] and NemA were optimized using the Structure Preparation module of MOE. The preparation procedure included the addition of hydrogens, the deletion of solvents, and adjustments of the formal charges. For the two protein models, the FMN cofactor was manually reduced to FMNH^-1^ by including a -1 charge at the N_1_ position and protonating at N_5_ [31,32]. In the case of NemA, it was ensured that the epsilon nitrogen of His182 and the delta nitrogen of His185 were protonated as the equivalent residues are believed to be important for substrate interaction in the *Bacillus subtilis* homologue YqjM [31].

### Molecular docking

The ligands (*trans*-2-hexenal, *trans*-2-hexenoic acid, *trans*-2-hexenedioic acid, or 6-amino-*trans*-2-hexenoic acid) were individually docked to the prepared receptor (1OYB or NemA) using the Dock module of MOE. The combination of four ligands and two receptors gave a total of 90 docking poses (S1 Table). A pharmacophore was placed in the 1OYB model to allow substrates to accept hydrogen bonds from His191 and Asn194. A similar pharmacophore was placed in the NemA model to accept hydrogen bonds from His182 and His185. Each pose of the docking entries was subsequently subjected to energy minimization using the Amber10 force field with extended Hückel theory in MOE. Molecular mechanics with generalized Born surface area (MMGBSA) calculation was carried out using Prime module of Maestro Ver. 10. The linear interaction energy (LIE) was obtained using the following equation:

E(LIE)=E_inter(complex)‑E_inter(water) (1)

where E_inter(complex) is the binding energy of ligand and enzyme, and E_inter(water) is the interaction energy between ligand and solvent. Both energy terms are negative values. A negative value in LIE indicates stronger interaction with protein than with aqueous solution.

### Chemicals

*Trans*-2-hexenal and *trans*-2-hexenoic acid were purchased from Sigma-Aldrich (St. Louis, MO, USA; 6-amino-*trans-*2-hexenoic acid was purchased from Toronto Research Chemicals (Brisbane, Canada) and *trans*-2-hexenedioic acid was synthesized by olefin metathesis by condensing acrylic acid and 4-pentenoic acid in the presence of 2^nd^ generation Grubbs catalyst. For details see S1 Supporting information. The free Gibbs energy was obtained by Group contribution method [33,34].

### Plasmid and strain construction

The plasmids used in this study are described in Table 1. Genes subcloned in the plasmids were synthesized *via* PCR amplification using DreamTaq DNA polymerase (Thermo Fisher Scientific, Waltham, MA, USA) and a standard PCR protocol, according to the manufacturer’s instructions. The gene *nemA*, from *E*. *coli* K12 MG1655, was amplified by PCR from a genomic DNA preparation extracted from *E*. *coli* K12 MG1655. The primers used contained the *Bam*HI restriction site (*nemA*_fw 5’-*CC****GGATCC***ATGTCATCTGAAAAACTG-3’) and the *Hin*dIII restriction site (nemA_rv 5’-*CCC****AAGCTT***TTACAACGTCGGGTAATC-3’). The *nemA* gene was then further subcloned into the pET28a plasmid under the control of T7 promoter (Merck Millipore, Billerica, MA, USA). The gene *OYE1*, coding for Oye1 (UniProt Q02899), of *S*. *pastorianus* was a kind gift from Neil C. Bruce (Department of Biology, University of York, UK) [35]. The gene *OYE1* was amplified by PCR. The primers used contained the *Bam*HI restriction site (*OYE1*_fw 5’-*CC****GGATCC***ATGTCATTTGTAAAAGATTTTAAGC-3’) and the *Sal*I restriction site (5’-*AGC****GTCGAC***TTACTTTTTGTCCCAGC-3’). The amplified products were inserted into the pQE30 plasmid under the control of T5 promoter (Qiagen, Hilden, Germany) as reported in Table 1. Each new plasmid construct was sequenced to verify the correctness of the subcloning and the absence of possible mutations (Eurofins Genomics, Ebersberg, Germany). The transformation of *E*. *coli* DH5α competent cells (Thermo Fisher Scientific) was performed according to the manufacturer’s instructions.

**Table 1:** Plasmids used in this study.

Plasmid	Features	Reference
pQE30	Amp^R^ + His-tag	Qiagen
pET28a	Kan^R^ + His-tag	Merck Millipore
pT7_OYE	Amp^R^ + His-tag	[35]
pET28a_n*emA*	Kan^R^ + *nemA* + His-tag	This study
pQE30_*OYE1*	Amp^R^ + *OYE1* + His-tag	This study

### Strains and media

The *E*. *coli* strains used in this work are described in Table 2. The *E*. *coli* strain K12 MG1655 was used for genomic DNA preparation. *E*. *coli* DH5α was used as an intermediate host for cloning, plasmid amplification, and maintenance, and was grown in LB medium with the addition of 1 g/L of glucose. Recombinant *E*. *coli* strains were selected on solid LB medium containing 2% (w/v) agar and 1 g/L of glucose. Plasmid preservation and selection were ensured by adding 100 mg/L ampicillin or 25 mg/L kanamycin to the LB medium, according to strain requirements. The *E*. *coli* strains BL21(DE3) (Merck Millipore) and NM522 were used for protein production.

**Table 2:** *E*. *coli* strains used in this study.

Strain	Reference
K12 MG1655	[36]
DH5α	[37]
BL21(DE3)	[38]
NM522	[39]
DH5α_pQE30_*nemA*	This study
BL21(DE3)_pET28a_*nemA*	This study
DH5α_pQE30_*OYE1*	This study
NM522_pQE30_*OYE1*	This study

### Protein production and purification

NemA and Oye1 were produced as fusion proteins, fused to an N-terminal His tag that was used for purification. NemA was produced in *E*. *coli* BL21(DE3) grown in auto-induction medium [40] containing 25 mg/L kanamycin for 16–18 hours at 37°C and 150 rpm. Oye1 was produced in *E*. *coli* NM522 grown in LB medium containing 100 mg/L ampicillin at 37°C and 150 rpm. When the optical density of the culture at 600 nm was around 0.6, 1 mM isopropyl-β-thiogalactopyranoside was added to the medium to induce the expression of *OYE1*. After induction, growth was continued at 30°C at 150 rpm for 16–18 hours. Cells of both BL21(DE3) pET28a_*nemA* and NM522_pQE30_*OYE1* were harvested by centrifugation (5525 x *g*, at 4°C for 20 min) and stored at –20°C until protein extraction. Cells were thawed and re-suspended in lysis buffer (50 mM potassium phosphate buffer, pH 7.4, 0.5 M NaCl, EDTA-free SIGMAFAST Protease Inhibitor Cocktail (Sigma-Aldrich)) and lysed by sonication (Branson Digital Sonifier, model 250) using a maximum amplitude of 30%, with “pulse on” for 0.7 s and “pulse off” for 1.0 s, for a total time of 20 s. Sonication was performed for 4 cycles, or until the solution became clear. The cells were kept on ice throughout the procedure. Cell debris was removed by centrifugation (5525 x *g*, at 4°C for 20 min) and the supernatant was filtered through a 0.45 μm nylon membrane (VWR, Radnor, PA, USA). The proteins of interest, NemA and Oye1, were purified on an ÄKTA purifier (GE Healthcare, Little Chalfont, UK) equipped with a HisTrap excel column (GE Healthcare) according to the manufacturer’s instructions, using 50 mM potassium phosphate buffer, pH 7.4. The enzymes were eluted using a linear gradient from 0 mM to 500 mM imidazole at a flow rate of 1 mL/min. The enzyme buffer was exchanged to 50 mM potassium phosphate buffer, pH 7.0, using Amicon Ultra centrifugal filters with a 10 kDa cut-off (Merck Millipore) according to the manufacturer’s instructions. The purity of the proteins was tested using SDS–page, and the concentration was determined at 280 nm using a NanoDrop 2000 full-spectrum UV-Vis spectrophotometer (Thermo Scientific). The extinction coefficient (ε_280_) and molecular weights (M) used to calculate the protein concentration were ε_280_ = 46870 M^-1^cm^-1^ and M = 43.1 kDa for NemA and ε_280_ = 73800 M^-1^cm^-1^ and M = 47.9 kDa for Oye1. The values of ε_280_ and M were retrieved from the web-based tool ProtParam available at ExPASy (https://web.expasy.org/protparam/). Purified enzymes were stored 50 mM potassium phosphate buffer (pH 7) at 4°C until use.

### Enzymatic activity assay

The activity of the purified enzymes NemA and Oye1 was assessed spectrophotometrically by monitoring the oxidation of NADPH at 340 nm in a FLUOstar plate reader (BMG Labtech, Ortenberg, Germany) with a 150 μL reaction volume. The standard enzyme reaction conditions were 6.67 μg/mL enzyme, 200 μM NADPH and 200 μM of the substrate of choice (i.e. *trans*-2-hexenal, *trans*-2-hexenoic acid, 6-amino-*trans-*2-hexenoic acid, or *trans*-2-hexenedioic acid) in 50 mM potassium phosphate buffer, pH 7.0 at 30°C. NADPH was added last to initiate the reaction. The reaction was monitored for 30 minutes every ca. 40 seconds. Prior to the addition of NADPH, the enzyme mix was incubated for 30 min at room temperature. The specific activity was calculated using a molar extinction coefficient of NADPH at 340 nm of 6200 L mol^-1^ cm^-1^.

### Inhibition test

The potential inhibition of the NemA and Oye1 by the substrates of interest was investigated by increasing the concentration of each compound while maintaining constant amounts of NADPH, natural substrate, and enzyme. NADPH was added after 30 min of incubation at room temperature, to allow for equilibrium to be reached between the natural substrate, potential inhibitors and the enzyme. The inhibition test was carried out two days prior to the enzymatic activity assay.

### NMR analysis

NMR spectra were recorded at 25°C on a Varian 400-MR 400 MHz spectrometer operating at 399.95 MHz for proton detection and at 100.58 MHz for carbon detection (S1–S5 Figs).

A concentration of 10 mg substrate in 0.7 mL D_2_O was used for the NMR measurements. A heat treatment study was performed to determine the stability and intramolecular reactivity of *trans*-2-hexenedioic acid and 6-amino-*trans-*2-hexenoic acid (S6 Fig). After NMR measurements on the samples the NMR tubes were placed in a water bath at 90°C, for 6 hours, after which they were cooled to room temperature and a second NMR spectrum was obtained.

### Ion chromatography analysis

The ion chromatography system Dionex^TM^ ICS-3000 (Thermo Fisher Scientific) equipped with an AminoPac PA-10 analytical column and AminoPac PA10 Guard column (Thermo Fisher Scientific) maintained at 30°C, was used for the detection of 6-amino-*trans-*2-hexenoic acid. The AAA-Direct Gradient Method 60/2, described in Dionex Application Note 150, was used [41]. Peak identities and quantification were confirmed by co-elution with standards.

## Results

### Identification of an alternative pathway to adipic acid from lysine

**Fig 2:**
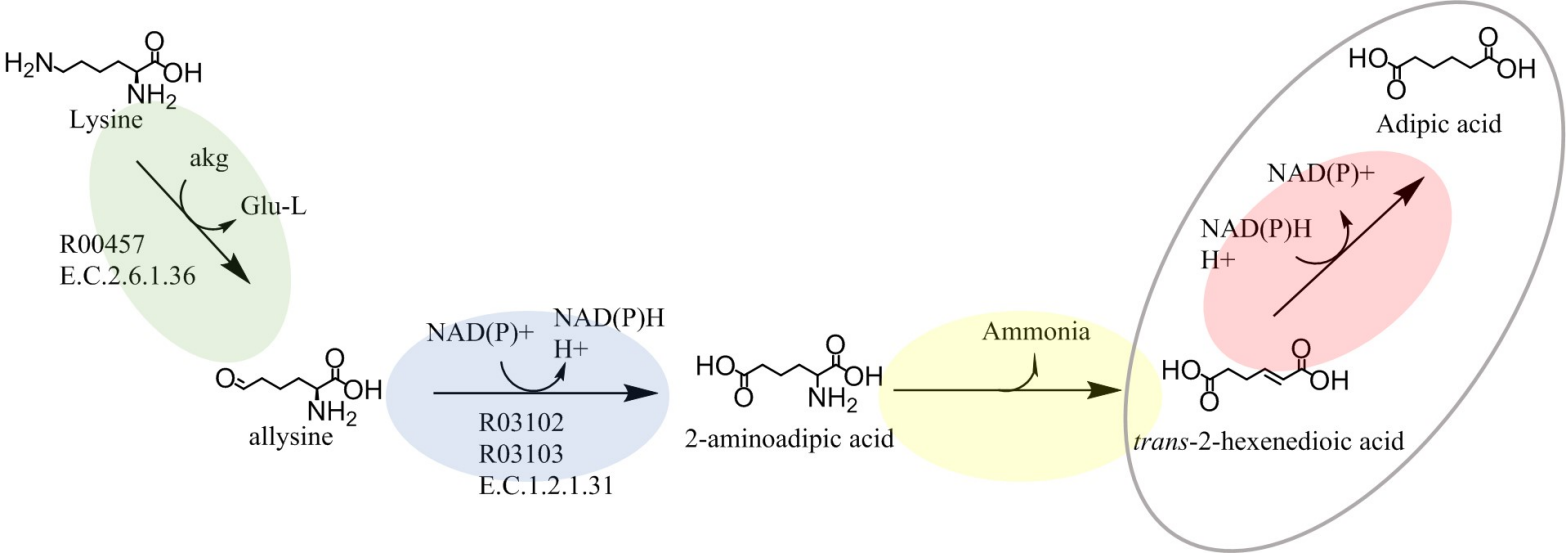
Metabolic pathways for the conversion of lysine to adipic acid. Our alternative pathway. Different types of enzymatic reactions are shown in different colors: removal of NH_2_ from α-carbon (yellow), reduction of unsaturated α,β bond (pink), removal of the terminal NH_2_ (green), and oxidation of aldehyde to carboxylic acid (blue). The enzymatic reactions targeted in this study are encircled. The reaction numbers according to the Kyoto Encyclopedia of Genes and Genomes are given for known reactions. The E.C. numbers according to the Braunschweig Enzyme Database are given for known enzymes. akg = alpha-ketoglutaric acid, Glu-L = L- glutamic acid.

In the pathway for the conversion of lysine to adipic acid [1] shown in Fig 1, enzymes capable of efficiently performing three out of four of the enzymatic reactions have not yet been identified. We investigated the pathway by careful data mining from the Kyoto Encyclopedia of Genes and Genomes, as well as from the Braunschweig Enzyme Database. In an attempt to circumvent the problem of having several unknown enzymes, we propose an alternative metabolic pathway (Fig 2), by changing the order of the chemical reactions. In this way, the number of unknown enzymes could be reduced without affecting the balanced redox potential of the pathway. The removal of the terminal NH_2_ group from 6-aminocaproic acid to form adipic acid semialdehyde (Fig 1) has not yet been observed to the best of our knowledge, whereas the removal of the terminal NH_2_ group from lysine by enzymatic activity to form allysine has been reported [42–45] (Fig 2). In addition, changing the order of the chemical reactions in the pathway will cause different intermediates to be formed, thereby increasing the number of potential substrates for a certain metabolic reaction (Fig 2). *trans*-2-Hexenedioic acid is now also included in the pathway (Fig 2), as well as 6-amino-*trans*-2-hexenoic acid (Fig 1), for the reduction of the unsaturated α,β bond. In fact, the reduction of the unsaturated α,β bond of the intermediate *trans*-2-hexenedioic acid is included in other suggested pathways for the production of adipic acid [1,46], indicating the importance of the reduction of this metabolite in the biosynthesis of adipic acid. However, both *trans*-2-hexenedioic acid and 6-amino-*trans*-2-hexenoic acid are rarely, if ever, found in nature, and it will thus probably be difficult to find enzymes with the desired specificity. The reduction of *trans*-2-hexenedioic acid using enoate reductases was recently reported [47], however the requirement for oxygen for an efficient bioprocess limits their use in both of the pathways considered here (Fig 2).

### Choice of substrates

**Fig 3:**
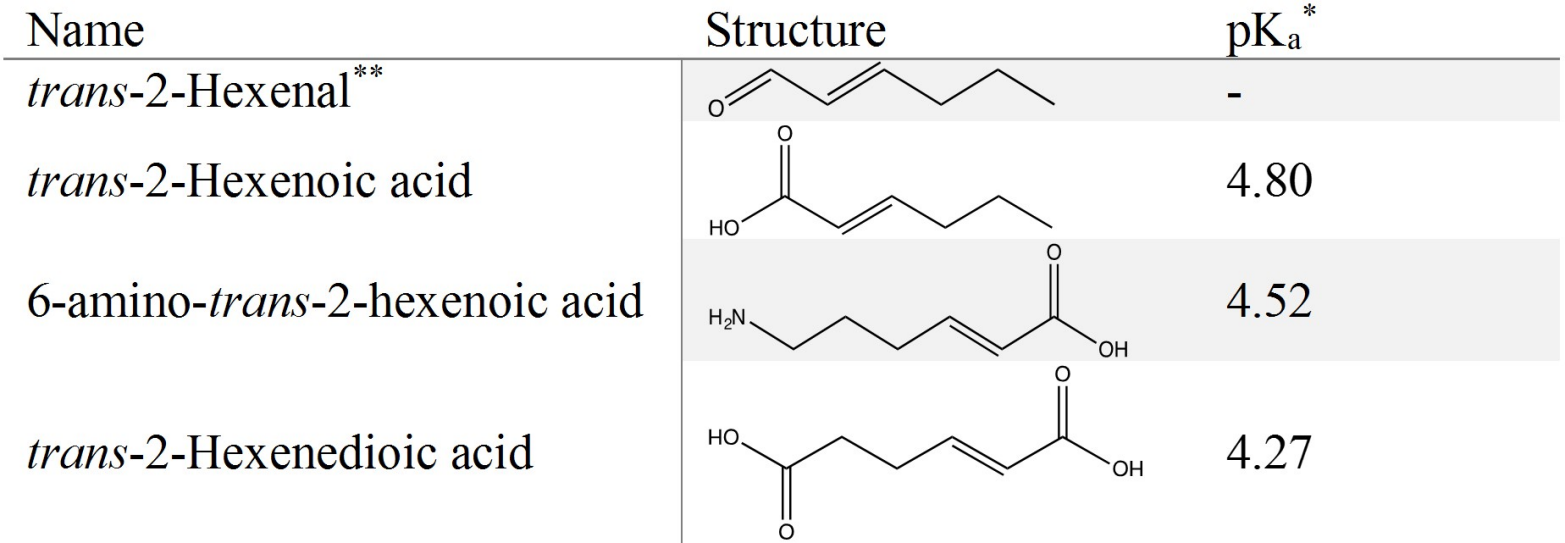
Substrates used for enzymatic activity determination in this study. *Obtained from scifinder.cas.org. For *trans*-2-hexenedioic acid the most acidic pK_a_ is given. ** Positive control.

In the present work, we focused on two enzymes, NemA from *E*. *coli* and Oye1 from *S*. *pastorianus*, as potential candidates for the reduction of the unsaturated α,β bond of 6-amino-*trans-*2-hexenoic acid and *trans*-2-hexenedioic acid, respectively. Both these enzymes are known to reduce *trans*-2-hexenal [35], which was included as a positive control. Since *trans*-2-hexenal is an aldehyde and the target substrates are carboxylic acids, *trans*-2-hexenoic acid was included as an additional substrate in order to better understand the ability of these two enzymes to reduce carboxylic substrates. The target substrates together with the positive control and *trans*-2-hexenoic acid are illustrated in Fig 3.

### *In silico* modelling

**Fig 4:**
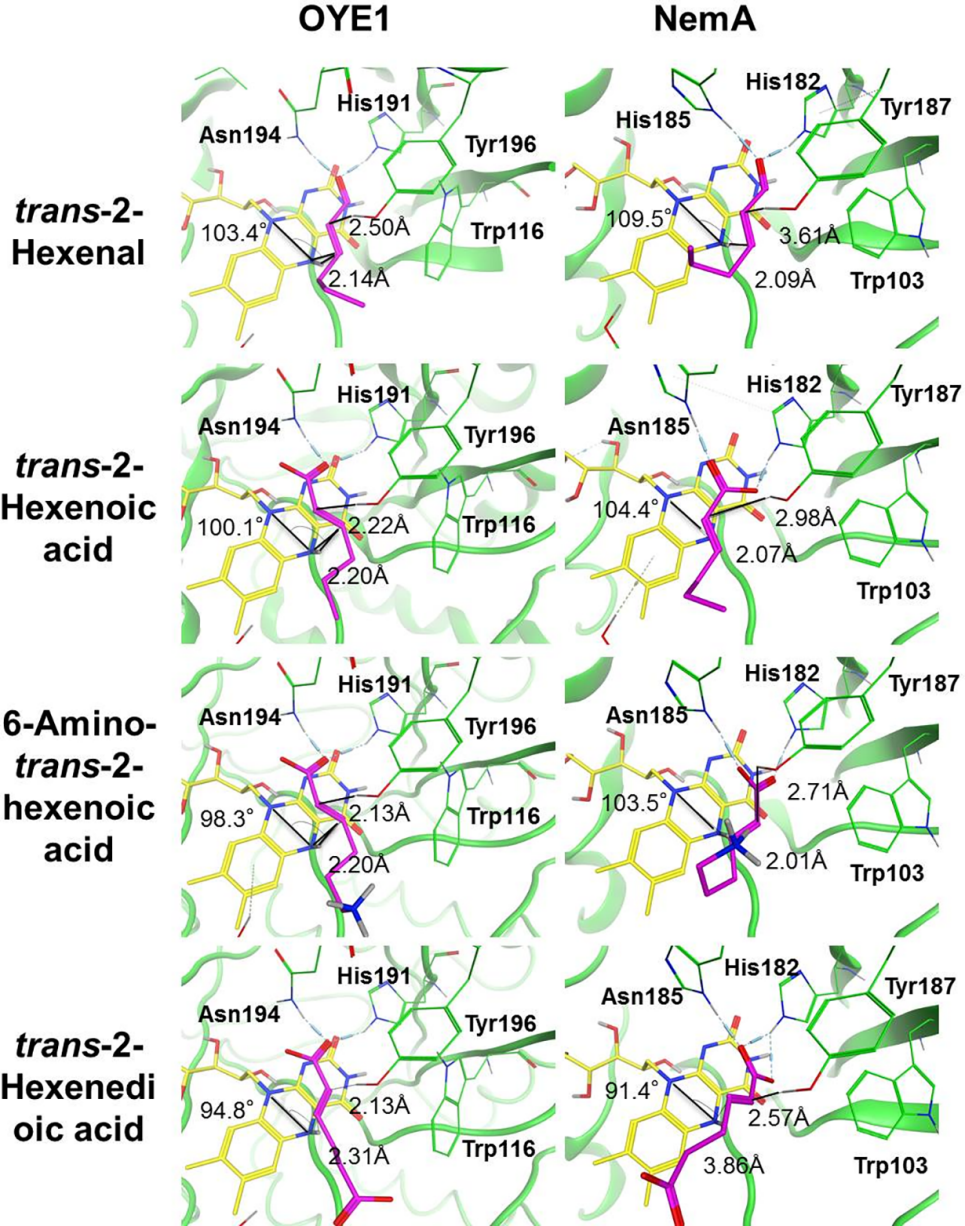
Molecular docking results to Oye1 and NemA for *trans*-2-hexenal (T2H), *trans*-2-hexenoic acid (T2HA), 6-amino-*trans-*2-hexenoic acid (6H2A), and *trans*-2-hexenedioic acid (H2EA). The distances between the α-carbon and hydroxyl hydrogen are indicated. Distances between the *β*-carbon and the N_5_ hydrogen of FMN are also shown. The angle is the N_10_-N_5_- *β* carbon angle. Measurements of the distances and angles are described in more detail in S7 Fig.

In order to explore possible interactions between NemA, Oye1 and the substrates of interest (Fig 3), molecular docking studies were performed (Fig 4). The publically available crystal structure 1OYB [25] of Oye1 was chosen (see Materials and methods). 1OYB has been used in previous computational studies of the interaction between Oye1 and various substrates, including α,β-unsaturated aldehydes [32], α,β-unsaturated ketoesters [48], and α,β-unsaturated ketones [48–50]. A homology model was constructed for docking studies on NemA, as no crystal structure is available.

Using the substrates of interest (*trans*-2-hexenal, *trans*-2-hexenoic acid, 6-amino-*trans-*2-hexenoic acid and *trans*-2-hexenedioic acid) and two receptors Oye1 and NemA, a total of 90 binding results were obtained (S1 Table). According to the literature, Oye1 can have two binding modes: normal and flipped (S8 Fig) [49–51]. Although the different binding modes result in different positions of the carbonyl carbon, the β-carbon is susceptible to hydride transfer regardless of binding mode, as long as it is within the vicinity of the N_5_ hydride of FMNH^-1^. Hence, different binding modes should not affect hydride transfer from FMNH^-1^ in the substrates considered here, while the distance between the hydride and β-carbon will have an effect. In addition, none of the substrates considered are ring structures, and they are not sterically hindered by Trp116, which is often mutated in order to introduce different binding modes with ring structures [51]. When substituted α,β-unsaturated substrates are reduced in the different binding modes, they result in different chirality [49]. We observed both normal binding and flipped binding modes (Fig 4, S8 Fig and S1 Table), but as the substrates considered here were not substituted at the α or β position, the same product was formed with both binding modes (i.e. no chirality was observed). Thus, no docking entry was rejected based on binding mode.

Binding of *trans*-2-hexenal to Oye1 showed that His191 and Asn194 formed hydrogen bonds with the carbonyl oxygen, as expected (Fig 4). In the case of NemA, His182 and His185 formed hydrogen bonds to the carbonyl oxygen. The highest scoring results were observed in the flipped binding mode for both Oye1 and NemA (S1 Table). The distances between the β-carbon of *trans*-2-hexenal and the N_5_ hydrogen of FMN were 2.14 Å and 2.09 Å, for Oye1 and NemA, respectively. The angles formed between N_10_, N_5_ and the β-carbon were 103.0° and 109.5° for Oye1 and NemA, respectively. These values are within the range (86.0°-111.8°) for bound substrates reported previously [49,52]. The best scoring results regarding the binding of *trans*-2-hexenoate to Oye1 or NemA were also found in the flipped mode, and showed two hydrogen bonds. In the case of Oye1, two-hydrogen bonding occurred on the same oxygen atom of the carboxyl group in *trans*-2-hexenoate from residues His191 and Asn194. For NemA, two-hydrogen bonding occurred separately on different oxygen atoms of the carboxyl group in *trans*-2-hexenoate from residues His182 and His185. The distances between the *β*-carbon of *trans*-2-hexenoate and the N_5_ hydrogen of FMN were 2.20 Å and 2.07 Å, for Oye1 and NemA, respectively, and the angles formed by N_10_, N_5_ and the *β*-carbon were 100.1° and 104.4°, respectively. Also in this case, the interactions between *trans*-2-hexenoate and Oye1 or NemA are within the ranges reported previously [49,52].

In the case of the substrate 6-amino-*trans-*2-hexenoic acid, a flipped binding mode was observed for 1OYB and the normal binding mode for NemA (Fig 4). The distances between the β-carbon of 6-amino-*trans-*2-hexenoic acid and the N_5_ hydrogen of FMN were 2.20 Å and 2.01 Å, for Oye1 and NemA, respectively. The angles formed between N_10_, N_5_ and the β-carbon were 98.3° and 103.5° for OYE1 and NemA, respectively. NemA formed two separate hydrogen bonds with two different carboxylic oxygen atoms, unlike Oye1. The binding mode to *trans*-2-hexenedioic acid was normal mode for both systems (Fig 4), with distances between the β-carbon of *trans*-2-hexenedioic acid and the N_5_ hydrogen of FMN of 2.31 Å for Oye1 and 3.86 Å for NemA. The angles formed by N_10_, N_5_ and *β* -carbon were 94.8° and 91.4°, for Oye1 and NemA, respectively. In the case of NemA, His182 was found to form two hydrogen bonds with separate carboxylate oxygen atoms and His185 formed a single hydrogen bond with one of the carboxylic oxygen atoms. The interactions of the target substrates with Oye1 and NemA are within the ranges described previously [49,52].

Based on the docking results presented above, both Oye1 and NemA seem to accommodate the substrates investigated. Although the docking results do not provide any insight into enzyme kinetics or reaction rates, they show that the enzymes are promiscuous enough to bind the substrates, and do not sterically hinder the binding interaction. However, in the case of *trans*-2-hexenedioic acid, it is notable that the docking results were often found in a way that the C6 carboxylic group, rather than the preferred C1 carboxylic group, interacted with the enzyme (S1 Table). If *trans*-2-hexenedioic acid interacts with the binding pocket in this orientation, the reaction will not occur unless the substrate re-orientates. None of the other substrates exhibited any orientation problems in docking. Based on the results obtained from docking studies, we decided to test the reactions *in vitro*.

### *In vitro* investigations

#### Protein production

**Fig 5:**
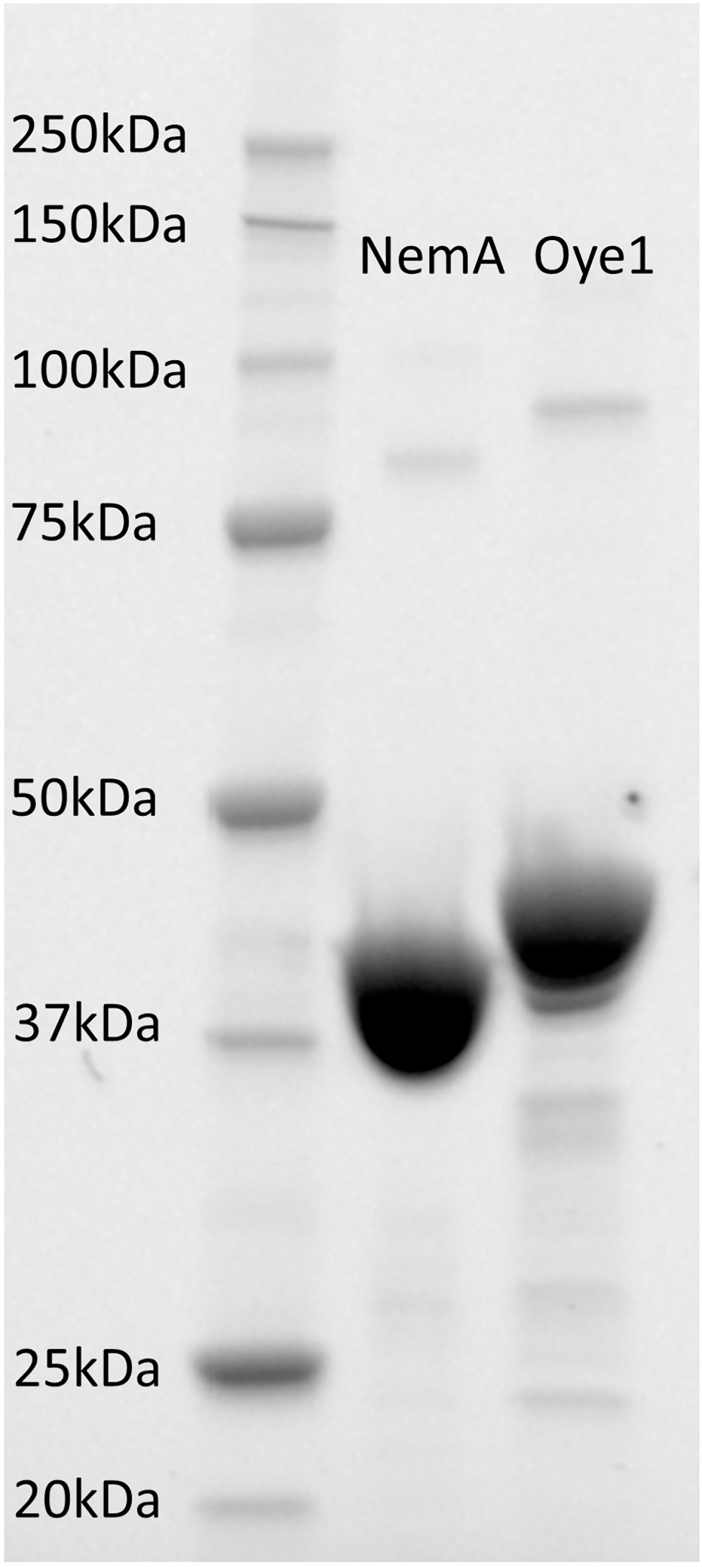
SDS–page separation of the purified enzymes NemA (43 kDa) and Oye1 (48 kDa).

After the expression of *nemA* and *OYE1* in *E*. *coli*, high, pure fractions of the enzymes were obtained upon affinity chromatography (Fig 5), namely 88.3 mg/ml and 26.6 mg/ml of NemA and Oye1, respectively. The total amount of protein obtained from 50 ml culture broth was ~30 mg for NemA and ~1 mg for Oye1.

#### Enzyme activity

**Fig 6:**
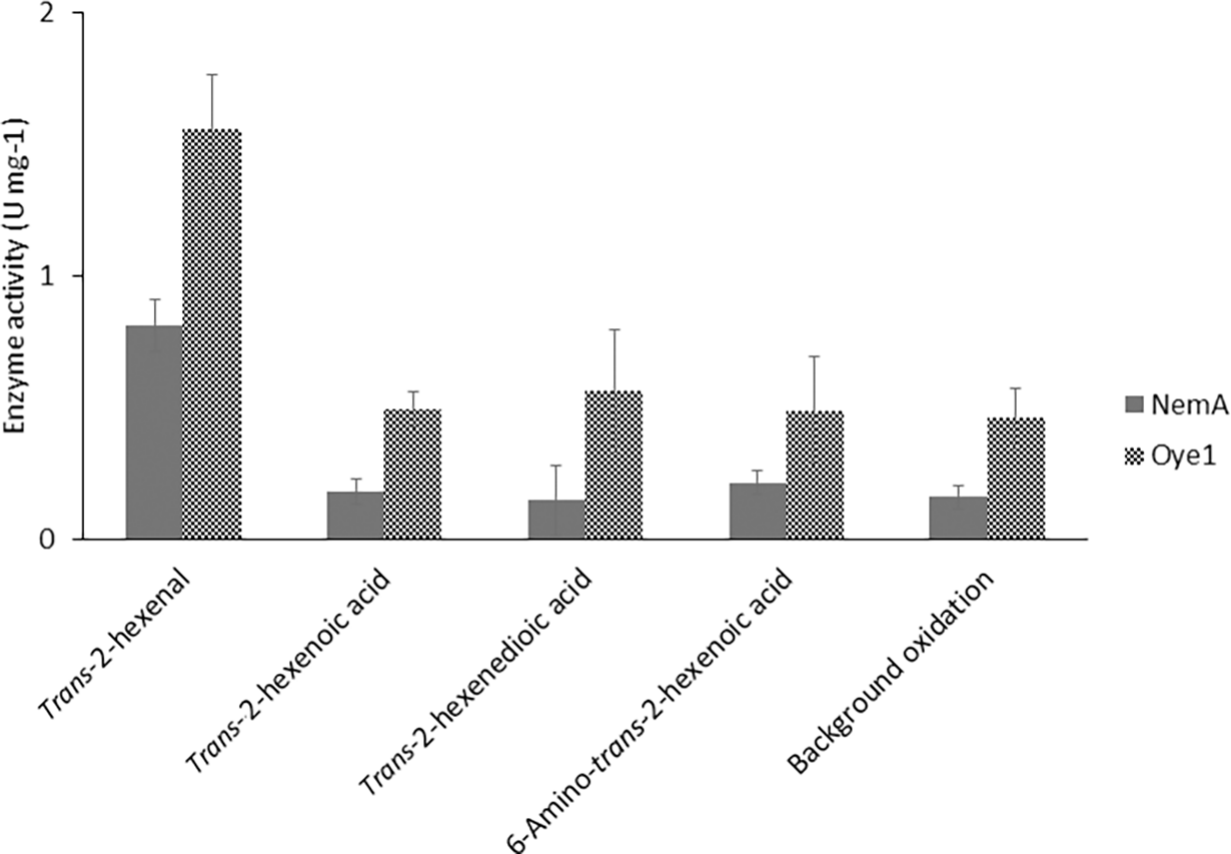
Specific activities of NemA and Oye1 on target substrates and controls. Units (U) are defined as μmol NADPH oxidized per min. All results are given as mean ± standard deviation of three replicates.

When testing the enzyme activity *in vitro* by monitoring NADPH oxidation, both enzymes showed activity on *trans*-2-hexenal that was significantly higher than the background oxidation of the co-factor. The background oxidation was measured as a control in a reaction mix with no addition of substrate, since molecular oxygen can act as a substrate for both NemA and Oye1. Although the enzymes were active on *trans*-2-hexenal, no activity was observed on the target substrates, or on *trans*-2-hexenoic acid (Fig 6). This supports previous findings that Oye1 cannot easily reduce mono-carboxylic substrates [27–29], and our experimental results suggest that this is also the case for NemA.

#### Test of the hypothesis of electron withdrawing potential

**Fig 7:**
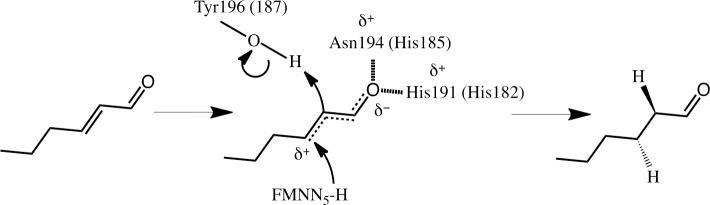
Proposed reaction mechanism of Oye1 and NemA, visualized for the reduction trans-2-hexenal. (Residues in brackets apply to NemA.) Upon hydrogen bonding of the aldehyde to the enzyme (hashed lines) electrons from the double bond are shifted towards the catalytic residues Asn194 (His182) and His191 (His185) (dotted lines), thereby creating a partial positive charge on the β-carbon (δ^+^) of the substrate, which activates the double bond and makes it prone to attack. When the double bond is activated the transfer of a hydride from the flavin N_5_ to the β-carbon of the substrate and protonation from Tyr196 (Tyr187) can occur, resulting in hexanal as the final product. The movement of electrons involved in the hydride attack and protonation are indicated by the curved arrows.

From the docking study, it was observed that both NemA and Oye1 can accommodate the target substrates and *trans*-2-hexenoic acid in their catalytic sites. Despite this, no experimental evidence of activity could be observed. One explanation of the difference in Oye1 reducing activity on aldehydes and ketones [53] and carboxylic acids, can be attributed to the difference in their electron withdrawing potential at neutral pH [54]. Due to the electron withdrawing potential of the oxygen in aldehydes and ketones, electrons are attracted from the neighboring carbon, creating a partial positive (δ^+^) charge on that carbon. Upon hydrogen bonding with the enzyme, the withdrawing potential increases further, and electrons from the unsaturated α,β bond are shifted towards the catalytic residues Asn194 and His191 in the case of Oye1 (His182 and His185 for NemA), thus making the β-carbon of the substrate more prone to attack, activating the double bond. When the double bond is activated the transfer of a hydride from the flavin N_5_ to the β-carbon of the substrate occurs allowing protonation from Tyr196 (Oye1) (Tyr187 for NemA) to occur [55] (Fig 7).

**Fig 8:**
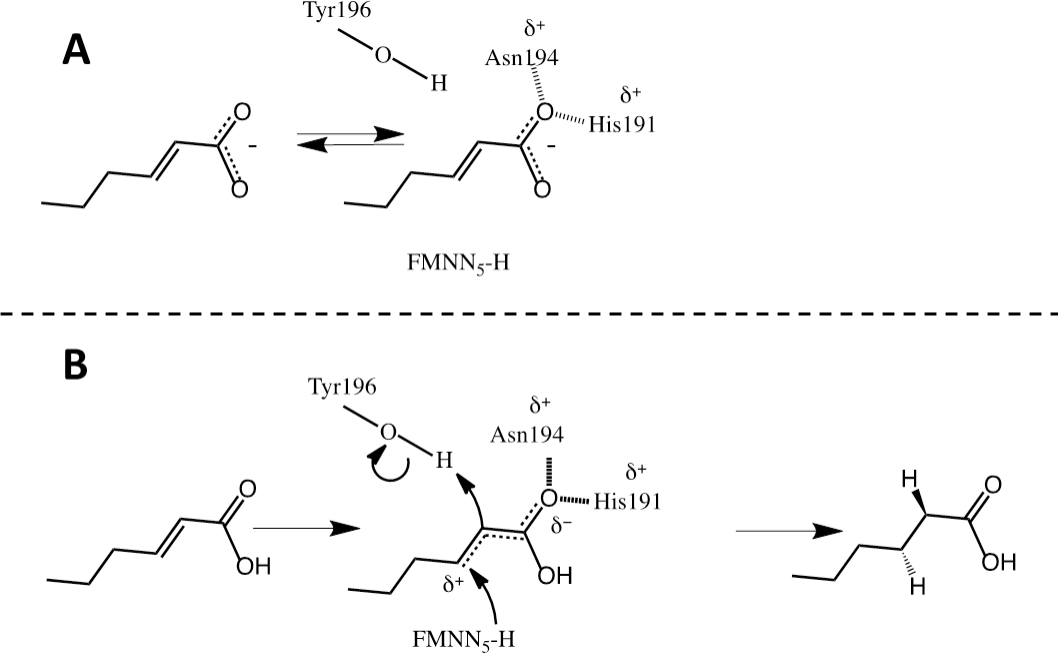
**Proposed reaction mechanism for Oye1 on** α,β **unsaturated carboxylic acid, here visualized with *trans*-2-hexenoic acid when the carboxylic acid is deprotonated (A) and when it is protonated (B)**. (A) The electrons of the additional negative charge are distributed between the two oxygens in a resonance structure (dotted lines). Upon hydrogen bonding with the catalytic residues of the enzyme, the electrons in the resonance structure will prevent activation of the unsaturated α,β bond and no reaction will occur. B) Upon hydrogen bonding to the enzyme (hashed lines) electrons from the double bond are shifted towards the catalytic residues Asn194 and His191 (dotted lines), thereby creating a partial positive charge (δ^+^) on the β-carbon of the substrate, which activates the double bond and makes it prone to attack. When the double bond is activated the transfer of a hydride from the flavin N_5_ to the β-carbon of the substrate and protonation from Tyr196 can occur, resulting in hexanoic acid as the final product. The movement of electrons involved in the hydride attack and protonation are indicated by the curved arrows.

At neutral pH, carboxylic acids are deprotonated, in contrast to aldehydes and ketones, and do not have an electron withdrawing potential. The lack of withdrawing potential is due to the fact that the electrons of the additional negative charge on the carboxylate group are distributed between the two oxygens in a resonance structure. Thus, electrons from the unsaturated α,β bond are prevented from being shifted towards the catalytic residues, and there is no reaction (Fig 8A). Protonated carboxylic acids, on the other hand, do not have a resonance structure, and thus have an electron withdrawing potential. It was therefore hypothesized that lowering the pH would make the carboxylic substrates more prone to react with the enzyme (Fig 8B).

The enzymatic reaction was tested experimentally between pH 3 and pH 6. At pH 3, which is well below the pKa of the substrates (Fig 3), no enzymatic activity was observed, even on the positive control *trans*-2-hexenal. As the enzyme did not show any activity at such a low pH, the hypothesis based on the withdrawing potential of protonated carboxylic acid could not be verified. The lowest pH at which enzymatic activity was observed on *trans*-2-hexenal was pH 5 for Oye1 and pH 6 for NemA. At these levels of pH, no activity could be observed on either of the target substrates, 6-amino-*trans-*2-hexenoic acid or *trans*-2-hexenedioic acid, or *trans*-2-hexenoic acid.

#### Possible inhibitory effects of the substrates

**Fig 9:**
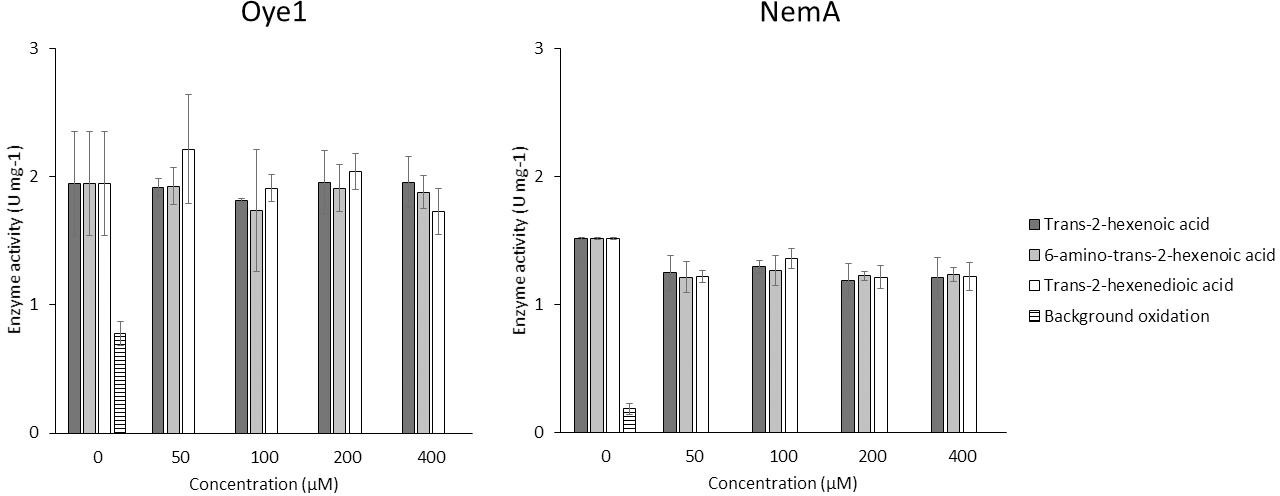
Inhibition test. Activity of NemA and Oye1 in the presence of *trans*-2-hexenoic acid, 6-amino-*trans*-2-hexenoic acid, and *trans*-2-hexenedioic acid investigated for their inhibitory effect at concentrations up to 400 μM. Results are given as mean ± standard deviation of three replicates.

Despite the observations in the docking studies that both enzymes could accommodate the target substrates 6-amino-*trans-*2-hexenoic acid and *trans*-2-hexenedioic acid, as well as *trans*-2-hexenoic acid in their active sites, no reactivity was observed. Therefore, it was hypothesized that these substances could act as direct inhibitors occupying the active site, thereby preventing the enzyme from reducing the control substrate *trans*-2-hexenal. To test this hypothesis, experiments were performed with fixed equimolar concentrations of *trans*-2-hexenal and the cofactor NADPH with increasing concentrations of 6-amino-*trans-*2-hexenoic acid, *trans*-2-hexenedioic acid or *trans*-2-hexenoic acid. No significant inhibition of the activity was observed, even at twofold higher concentrations of potential inhibitors compared to *trans*-2-hexenal. The activity of NemA seems to be somewhat lower in the presence of potential inhibitors, but no trend was observed with increasing concentrations of the potential inhibitors (Fig 9).

#### Possible binding of *trans*-2-hexenal and *trans*-2-hexenoic acid to the surface of the enzyme

Since *trans*-2-hexenoic acid was not reduced by Oye1 or NemA, the possibility of an electrostatic interaction between *trans*-2-hexenal and *trans*-2-hexenoic acid and the enzyme surface was investigated. To this end, the electrostatic potential was mapped onto the surface of Oye1 (S9 and S10 Figs) and NemA (S11 and S12 Figs) with the aim of identifying regions of positive potential capable of interacting strongly with the negatively charged carboxylates. One such strong positive region in Oye1 was identified containing Arg334 and Lys338 (Arg131 and Arg143 in the case of NemA), to which *trans*-2-hexenal and *trans*-2-hexenoate were docked. The positively charged region was located on the back side of Oye1, while it was located very close to the catalytic site in NemA. Molecular docking results showed that the Arg334 and Lys338 positions of Oye1 were able to form hydrogen bonds with the carboxylate function of *trans*-2-hexenoate with a docking score of -4.1881, while that of *trans*-2-hexenal was -4.0934. The Arg143 and Arg131 positions of NemA formed hydrogen bonds with the carboxylate of *trans*-2-hexenoate, with a docking score of -3.2458, and with *trans*-2-hexenal with a docking score of -3.0907. Using MMGBSA, the binding energies of the respective complexes were computed [56]. For *trans*-2-hexenal, the ligand preferentially binds to the catalytic site of Oye1 over binding to the identified positive patch on the protein surface, by 22.5 kcal/mol. For *trans*-2-hexenoate, the situation is the opposite in that the acid interaction with Lys338-Arg334 is lower by 14.8 kcal/mol compared to binding in the catalytic site of Oye1. *trans*-2-Hexenal also preferentially bound to the catalytic site of NemA over the positive patch on enzyme surface by 22.2 kcal/mol, whereas *trans*-2-hexenoate interaction with Arg143-Arg131 on NemA surface was lower by 8.3 kcal/mol compared to catalytic site of NemA. Additionally, using linear interaction energy estimations, the interaction energy of the substrate with the catalytic pocket was compared to the interaction energy with water [57]. *trans*-2-Hexenal preferentially bound to the pocket of Oye1 and NemA with linear interaction energy of -39.44 kcal/mol and -34.25 kcal/mol, respectively. However, *trans*-2-hexenoate preferentially interacted with water than Oye1 and NemA with linear interaction energy of 11.91 kcal/mol and 8.36 kcal/mol, respectively (see Eq 1 for definition of LIE). Since *trans*-2-hexenoate does not prefer to stay in the catalytic pocket, this might provide a possible explanation for the lack of catalysis or inhibition in these studies.

#### Heat sensitivity of 6-amino-*trans-*2-hexenoic acid and *trans*-2-hexenoic acid

**Fig 10:**
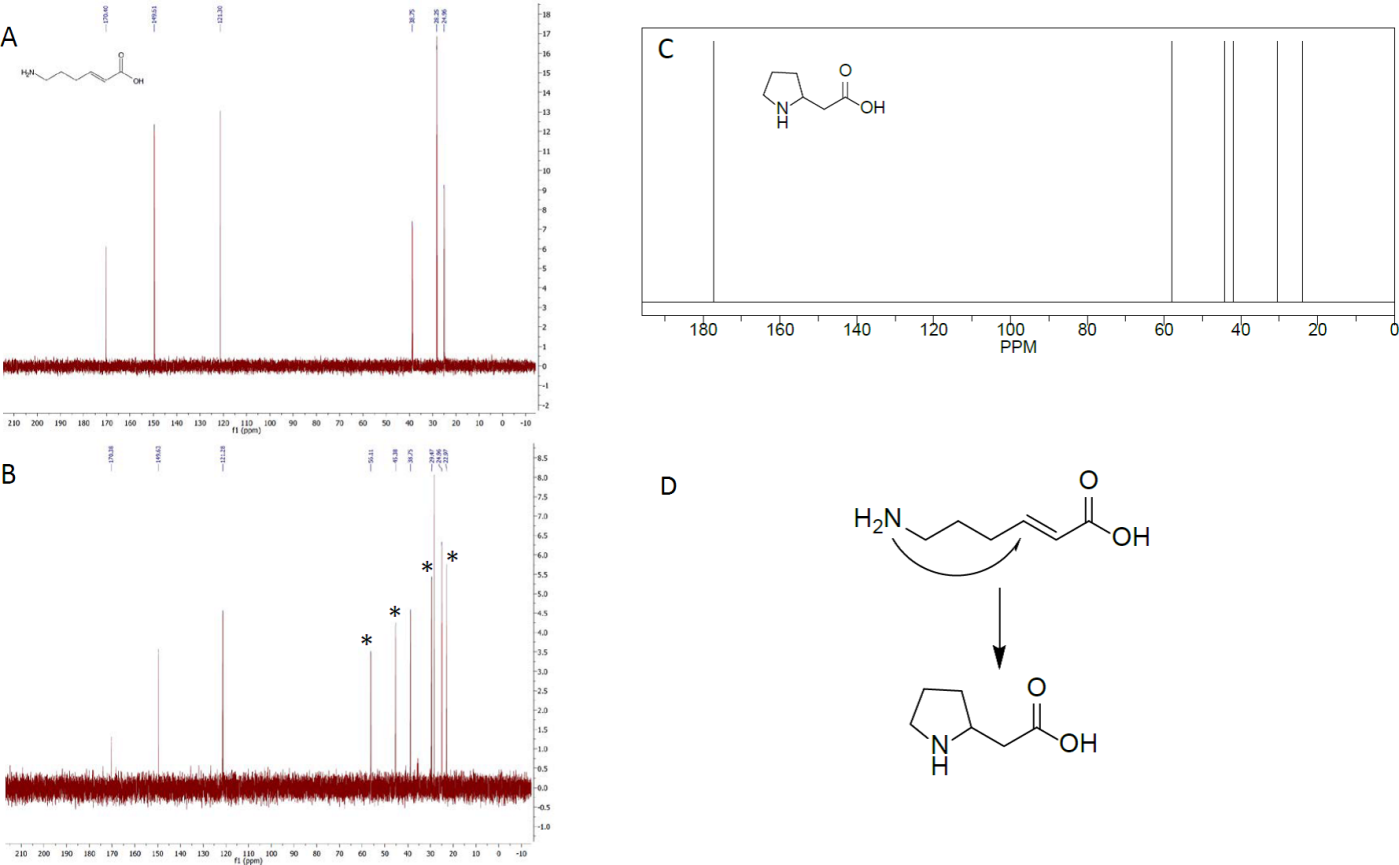
Identification of ring closure product of 6-amino-*trans-*2-hexenoic acid. NMR spectra of A) 6-amino-*trans-*2-hexenoic acid at 25°C before heating, B) 6-amino-*trans-*2-hexenoic acid after being heated at 90°C for 6 h, where additional peaks can be seen, indicated by asterisks. C) NMR spectrum of 2-pyrrolidineacetic acid. D) Proposed 2-pyrrolidineacetic acid formation mechanism by intra-molecular cyclization.

From studying the structures of the target substrates, we realized that 6-amino-*trans-*2-hexenoic acid might be prone to internal ring closure, forming 2-pyrrolidineacetic acid. If this is the case, this could explain why no activity was observed on this substrate. When analyzing 6-amino-*trans-*2-hexenoic acid by chromatographic methods, after incubation at 30°C for 4 hours, an unknown peak was indeed observed (Panel A in S6 Fig). The unknown peak was not observed in the control sample, which had not been incubated at 30°C (Panel B in S6 Fig). When analyzing boiled samples of 6-amino-*trans-*2-hexenoic acid the 6-amino-*trans-*2-hexenoic acid peak disappeared completely, and instead an unknown peak emerged (Panel B in S6 Fig). NMR measurements revealed that an additional substrate was indeed formed upon heating of 6-amino-*trans-*2-hexenoic acid. When comparing the NMR spectra obtained with spectra from NMR prediction software the additional substrate was found to be most likely 2-pyrrolidineacetic acid (Fig 10), formed through an intra-molecular Michael addition. The formation of pyrrolidine from amines by an intra-molecular reaction has been reported in the literature [58]. While the presence of 6-amino-*trans-*2-hexenoic acid did not inhibit the enzymatic activities (Fig 9), the fact that 6-amino-*trans-*2-hexenoic acid spontaneously undergoes intramolecular cyclization *in vitro* should be taken into consideration when the previously reported pathway [1] is discussed. No intramolecular cyclization was observed when *trans*-2-hexenedioic acid was heated to 90°C for 6 hours, but only a minor *trans*-*cis* isomerization of less than 5% (S3 Fig). The lack of intra-molecular cyclization of *trans*-2-hexenedioic acid was expected since the carboxylate is a much weaker nucleophile than amines.

## Discussion

In the present work, we have investigated if and how the pathway proposed by Burgard *et al*. [1] for the conversion of lysine into adipic acid could be realized. The main challenge associated with this pathway is the lack of efficient enzymes for three of the four enzymatic reactions. To circumvent this problem, we proposed an alternative pathway, in which the order of the chemical reactions was changed. In doing this, we were able to reduce the number of unknown enzymatic steps, and include an additional substrate for each type of chemical reaction, without affecting the balanced redox potential of the pathway (Fig 2). We also set out to identify important factors in the efficient reduction of the unsaturated α,β bonds of the pathway-specific intermediates 6-amino-*trans-*2-hexenoic acid and *trans-*2-hexenedioic acid, under aerobic conditions. Aerobic conditions are required to maximize the yields of the selected pathways [1], however efficient reduction of unsaturated α,β bonds in carboxylates under aerobic conditions has previously proven difficult [27–29]. Our aim was to gain detailed knowledge on the reaction, and the enzymes NemA and Oye1 were investigated with regard to their potential to reduce the unsaturated α,β bond of 6-amino-*trans*-2-hexenoic acid and *trans*-2-hexenedioic acid both theoretically and experimentally.

According to the results obtained from the *in silico* modelling, both target substrates, 6-amino-*trans*-2-hexenoic acid and *trans*-2-hexenedioic acid, and the carboxylic substrate *trans*-2-hexenoic acid could fit in the enzymatic pocket of both Oye1 and NemA. In the case of Oye1 two hydrogen bonds were observed with one of the oxygen atoms in the carboxylate group with the known substrate-binding residues Asn194 and His191 [25]. NemA also created two hydrogen bonds, but separately, with one hydrogen bond to each of the oxygens of the carboxylate group, one with residue His182 and the other with His185 (Fig 4). Despite the difference in hydrogen bonds, the distance between the N_5_ of FMN and the β-carbon of both target substrates and *trans*-2-hexenoic acid, as well as the angle formed by N_10_/N_5_ and the β-carbon were within the range necessary for the reaction to take place with both proteins [49,52], indicating a reaction favoring binding. Despite the positive binding predicted by *in silico* modelling, no activity was observed *in vitro* on either of the target substrates or the carboxylic substrate *trans*-2-hexenoic. These results are consistent with those in a recent study in which *in vitro* results for *trans*-2-hexenoic acid were negative for YqjM, an Oye family member, despite a successful *in silico* docking experiment [59]. While this does not explain the negative *in vitro* observations, it shows that successful docking results do not necessarily mean successful *in vitro* results.

The fact that no activity was observed experimentally may be partly due to a lack of insight into whether hydride transfer to the β-carbon actually takes place, and if it does, how rapid the reaction proceeds. For the reaction to occur, the unsaturated α,β bond must be activated. Aldehydes and ketones have electron withdrawing potential which, upon hydrogen bonding with the enzyme, will increase further and activate the unsaturated α,β bond (Fig 7). Carboxylic acids, on the other hand, do not have any electron withdrawing potential at neutral pH, and are thus unable to activate the unsaturated α,β bond (Fig 8A). The difference in electron withdrawing potential was not considered in the present docking experiments, but we attempted to induce electron withdrawing potential of the carboxylate group by protonating the acid by lowering the pH. However, the enzymes lost their catalytic activity completely at pH below the pKa of the target substrates and *trans*-2-hexenoic acid.

**Fig 11:**
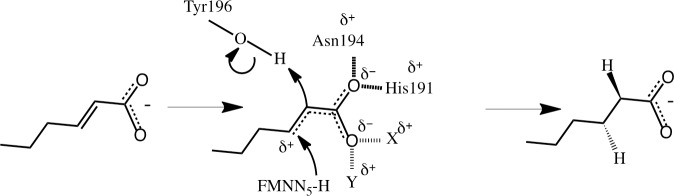
Proposed mechanism for reduction of deprotonated carboxylic acid by Oye1. Engineering of the enzyme by substitution of the native residues with putative X and Y residues could lead to the formation of hydrogen bonds between the enzyme binding pocket and both oxygens of the carboxylate group. Upon hydrogen bonding to the enzyme (hashed lines) electrons from the double bond are shifted towards the catalytic residues Asn194 and His191 for one of the oxygens, and to the residues X and Y for the other oxygen (dotted lines), thereby creating a partial positive charge on the β-carbon (δ^+^) of the substrate, which activates the double bond, making it prone to attack. When the double bond is activated the transfer of a hydride from the flavin N_5_ to the β-carbon of the substrate and protonation from Tyr196 can occur, resulting in hexanoic acid as the final product. The movement of electrons involved in the hydride attack and protonation are indicated by the curved arrows. For simplicity, only the mechanism for Oye1 is shown.

An alternative way of increasing the electron withdrawing potential is by creating additional hydrogen bonds to the oxygens of the carboxylate group. If both oxygens of the carboxylate group form two hydrogen bonds each with the enzyme, this could induce an electron withdrawing potential, and the unsaturated α,β bond would be activated, favoring the reaction (Fig 11). In order to test this hypothesis, in-depth *in silico* studies should be carried out to identify protein engineering strategies to create such hydrogen bonds in the enzymes NemA and Oye1. The engineering strategy should also take into account differences in the electron withdrawing potentials of the catalytic residues. For instance, positively charged residues, such as protonated histidine, are more likely to attract electrons and favor reaction than polar residues, such as asparagine.

Although the results from docking studies suggested that the target substrates 6-amino-*trans*-2-hexenoic acid and *trans*-2-hexenedioic acid, together with *trans*-2-hexenoic acid, could fit in the active site and create hydrogen bonds with both NemA and Oye1, despite the fact no enzymatic activity could be observed, we suggest, based on computed LIE data, that these substrates have better interaction with water than the catalytic pocket. If the *trans*-2-hexenoic acid does not stay in the pocket long enough, catalysis might not take place on the enoate moiety. Since *trans*-2-hexenoic acid preferentially interact with water than the enzyme, it spends more time in the solution than in the catalytic pocket. While the aldehyde moiety in *trans*-2-hexenal is stabilized by hydrogen bonds within the catalytic pocket, the binding residues might not be efficiently stabilizing the negative charge on enoate on *trans*-2-hexenoic acid. Thus, engineering the catalytic pocket for further stabilizing the enoate moiety will be necessary.

Another possible reason why 6-amino-*trans*-2-hexenoic acid was not reduced, could be that this substrate spontaneously forms an internal ring structure leading to the formation of 2-pyrrolidineacetic acid upon warming. The compound 2-pyrrolidineacetic acid does not contain an unsaturated α,β bond and hence cannot serve as a substrate for NemA or Oye1. The finding that this substrate can form 2-pyrrolidineacetic acid upon warming, whereas *trans*-2-hexenedioic acid does not, favors our suggested alternative pathway rather than the one proposed by Burgard *et al*. (Fig 2). According to the *in silico* study, *trans*-2-hexenedioic acid can bind to the enzymes with either of its two carboxylate groups. Which position favors the reaction depends on which of the two carboxylates groups binds to the enzyme. The possibility of *trans*-2-hexenedioic acid binding with the catalytic pocket *via* either of its carboxylate groups, where one is in a suitable position for reaction whereas the other is not, will reduce the rate of the reaction, but not prevent it. However, the low reaction rate of the alternative pathway for the production of adipic acid from lysine must be addressed.

Despite our negative *in vitro* results, the previously reported activity of NemA on 6-amino-*trans*-2-hexenoic acid for reduction of the unsaturated α,β bond to yield 6-aminocaproic acid [22] indicates that NemA may be an interesting enzyme to target. However, the extremely low yield achieved (< 0.5% moles of product per mole substrate) after 48 hours of incubation, despite employing an assay with regeneration of NADPH, brings into question the feasibility of the enzyme for *in vivo* reactions. Nevertheless, NemA may be a suitable target for protein engineering, as discussed in this paper. Protein engineering of NemA could result in an enzyme able to catalyze the challenging reactions; namely the reduction of the unsaturated α,β bonds of 6-amino-*trans*-2-hexenoic acid and *trans*-2-hexenedioic acid, which are important steps towards the fulfillment of biobased production of adipic acid.

The α,β-reduction we aimed for here intrinsically requires removal of the α-amino group both in lysine and amino adipic acid. This is one of the difficult steps in the pathway especially because it simultaneously requires removal of non-acidic hydrogen at the β-position. Thermodynamics also does not favor releasing α-amino groups (S2 Table). Our targeted reaction has so far only been shown to be possible for certain aromatic amino acids, e.g., tyrosine or phenylalanine, where the β-position hydrogen can be abstracted [60]. Thus, it might be necessary to design a different pathway consisting of transamination of the α-amino group and subsequent reduction of the ketone group followed by dehydration to reach enoate moiety.

Moreover, the reduction reaction discussed in the present study highly resembles part of the chain elongation step in the fatty acid biosynthesis and reverse β-oxidation. During fatty acid biosynthesis, the acyl moieties are bound to acyl carrier protein (ACP) [61] while they are bound to coenzyme A during reverse β-oxidation [4,5]. Hence, the oxidoreductases under the enzyme classification 1.3.1. acting on enote moieties are active on ACP- or coenzyme A- activated substrates. ACP- or coenzyme A-independent oxidoreductases in the category are mostly oxygen sensitive [62]. Thus, in order to reduce the α,β-double bond in the enoate moieties, it might be necessary to activate the substrates with coenzyme A and subsequently release coenzyme A after the reduction has taken place.

## Conclusions

We have identified an alternative pathway for converting lysine to adipic acid, with a reduced number of unknown enzymes and maintained, balanced redox potential. In addition, we have identified several parameters important for the achievement of the enzymatic reduction of unsaturated α,β bonds of 6-amino-*trans*-2-hexenoic acid and *trans-*2-hexenedioic acid. We showed theoretically that the substrates could fit in the enzymatic pocket, and that increasing the electron withdrawing potential by protein engineering to create additional hydrogen bonds between both oxygens in the carboxylate group should improve the catalytic capability. We also suggest that residues that have high electron withdrawing potential for the creation of hydrogen bonds should be utilized in protein engineering strategies. We believe that this study has provided important knowledge that will be useful in realizing a metabolic pathway for biobased adipic acid production.

## Supporting information

S1 Table Pharmacophore-based docking results of *trans*-2-hexenal, *trans*-2-hexenoic acid, *trans*-2-hexenedioic acid, and 6-amino-*trans*-2-hexenoic acid with Oye1 (1OYB) and homology modeled NemA. Pose entry are listed in the order of increasing docking score for each substrate-enzyme pair. Lowest docking score implies the most stable interaction. Criterion of which the pose entry is rejected is highlighted under the corresponding column. Rejected entry is also highlighted under the result column. ^a^ Binding mode according to previous studies [49,51]. ^b^ Distance between beta carbon and N_5_ hydrogen measured after post-docking energy minimization. Distances below 3.96 Å were only accepted. The limit of distance between beta carbon and N_5_ (4.1 Å) [49] and the measured distance between N_5_ and N_5_ hydrogen (1.04 Å) were used to obtain 3.96 Å. ^c^ Distance between alpha carbon and Tyr196 hydroxyl hydrogen measured after post-docking energy minimization (Tyr187 for NemA). ^d^ The angle defined by N_10_-N_5_-beta carbon. Angle range of 86.0°-111.8° is accepted only [49,52].
(XLSX)

S2 Table Thermodynamic feasibility of reactions in pathways shown in this study. Theoretical change of free Gibbs energy in each reaction step is calculated using ChemDraw software and provided here (in kJ/mol).
(XLSX)

S3 Table Four exemplary docking calculation results for Oye1 (1OYB) and *trans*-2-hexenal. (DOCX)

S4 Table Four exemplary docking calculation results for Oye1 (1OYB) and *trans*-2-hexenoic acid. (DOCX)

S1 Supporting information Further details on material and methods. (DOCX)

S1 Fig ^1^H NMR (left) and 13C NMR (right) of *trans*-2-hexenedioic acid in D_2_O. (TIF)

S2 Fig 2D correlation spectroscopy of *trans*-2-hexenedioic acid in D_2_O showing the coupling patterns in the molecule. (TIF)

S3 Fig ^1^H NMR of *trans*-2-hexenedioic acid in D_2_O after heating at 90°C for 6 hours. *trans*-2-Hexenedioic acid was heated at 90°C for 6h. No intramolecular reaction was detected, only a small, less than 5%, isomerization from *trans* to cis was observed by new signals for the *cis*-alkene at 6.19, td, J = 12, 8 Hz, 5.71 dt, J = 12, 2 Hz.
(TIF)

S4 Fig ^1^H NMR (left) and 13C NMR (right) of 6-amino-*trans*-2-hexenoic acid in D_2_O. (TIF)

S5 Fig ^1^H NMR (left) and 13C NMR (right) of 6-amino-trans-2-hexenoic acid in D_2_O heated at 60°C for 6 hours. (TIF)

S6 Fig Ion Chromatographic separation of 6-amino-*trans*-2-hexenoic acid. A) 100 μM 6-amino-*trans*-2-hexenoic acid, treated at 30°C for 4 hours. B) 100 μM 6-amino-*trans*-2-hexenoic acid 100 μM (not boiled) (blue) and 6-amino-*trans*-2-hexenoic acid 100 μM boiled (orange).
(TIF)

S7 Fig Exemplary distance and angle measurements in docking results. Distance between hydroxyl hydrogen of Tyr196 of Oye1 (Tyr187 for NemA) and alpha carbon is measured. Distance between N_5_ hydrogen and the beta carbon is also measured (threshold 3.96 Å). The angle is defined by N_10_-N_5_-beta carbon. The accepted range for this angle is 86.0° - 111.8°.
(TIF)

S8 Fig Representative decision criteria for binding mode. (A) Illustration of normal binding mode (magenta) and flipped binding mode (green). Coordinates are extracted from previously described reports (PDB = 4GWE and 4GE8) [51]. (B) Determination of binding mode according to the C1 position respect to α and β carbons. (C) Exemplary normal binding result for *trans*-2-hexenal (cyan). (D) Exemplary flipped binding mode for *trans*-2-hexenal (yellow).
(TIF)

S9 Fig Electrostatic interaction of Oye1 and *trans*-2-hexenal. Potential interaction with positive patch on enzyme surface and substrate is shown. A) Overall electrostatic view of 1OYB with bound *trans*-2-hexenal on the 1OYB surface. Yellow arrow indicates the relative position of the electrostatic interaction. B) Zoomed-in view of the interaction of bound *trans*-2-hexenal (yellow) and the 1OYB enzyme. C) Hydrogen bonding of Lys338 and Arg334 to carbonyl group of *trans*-2-hexenal.
(TIF)

S10 Fig Electrostatic interaction of Oye1 and *trans*-2-hexenoate. Potential interaction with positive patch on enzyme surface and substrate is shown. A) Overall electrostatic view of 1OYB with bound *trans*-2-hexenoate on the 1OYB surface. Yellow arrow indicates the relative position of the electrostatic interaction. B) Zoomed-in view of the interaction of bound *trans*-2-hexenoate (yellow) and the 1OYB enzyme. C) Hydrogen bonding of Lys338 and Arg334 to carboxylate group of *trans*-2-hexenoate.
(TIF)

S11 Fig Electrostatic interaction of NemA and *trans*-2-hexenal. Potential interaction with positive patch on enzyme surface and substrate is shown. A) Overall electrostatic view of NemA with bound *trans*-2-hexenal on the NemA surface. Yellow arrow indicates the relative position of the electrostatic interaction. Green arrow indicates the catalytic pocket. B) Zoomed-in view of the interaction of bound *trans*-2-hexenal (yellow) and the NemA enzyme. C) Hydrogen bonding of Arg131 and Arg143 to carbonyl group of *trans*-2-hexenal.
(TIF)

S12 Fig Electrostatic interaction of NemA and *trans*-2-hexenoate. Potential interaction with positive patch on enzyme surface and substrate is shown. A) Overall electrostatic view of NemA with bound *trans*-2-hexenoate on the NemA surface. Yellow arrow indicates the relative position of the electrostatic interaction. Green arrow indicates the catalytic pocket. B) Zoomed-in view of the interaction of bound *trans*-2-hexenoate (yellow) and the NemA enzyme. C) Hydrogen bonding of Arg131 and Arg143 to carboxylate group of *trans*-2-hexenoate.
(TIF)

S13 Fig Representative ligand interaction for *trans*-2-hexenal docking (left) and *trans*-2-hexenoate (right) with 1OYB. Green arrows indicate hydrogen bonding between the enzyme and substrate.
(TIF)
