# Supplementary material for: In silico and in vitro studies of the reduction of unsaturated α,β bonds of trans-2-hexenedioic acid and 6-amino-trans-2-hexenoic acid – Important steps towards biobased production of adipic acid
Source: PLoS One. 2018 Feb 23;13(2):e0193503. doi: 10.1371/journal.pone.0193503 (PMC5825115; doi:10.1371/journal.pone.0193503)
Supplement: S3 Table — (DOCX) [file pone.0193503.s003.docx]

**S2** **Table**. **Four exemplary docking calculation results for Oye1 (1OYB) and *trans*-2-hexenal.**

| 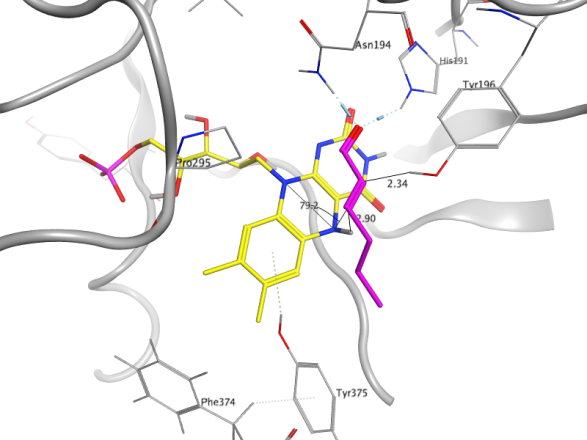 | 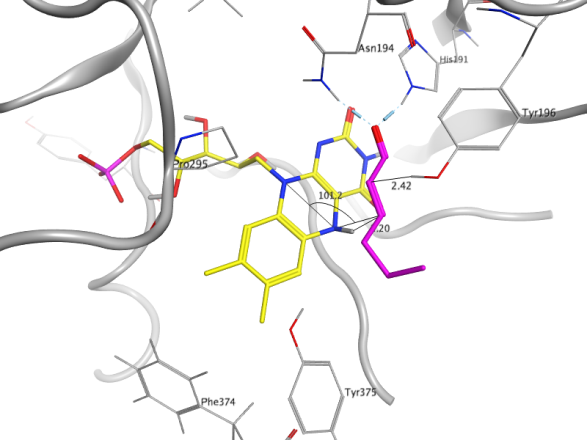 |
| --- | --- |
| Pose entry 2  Rejected | Pose entry 3  Accepted |
| 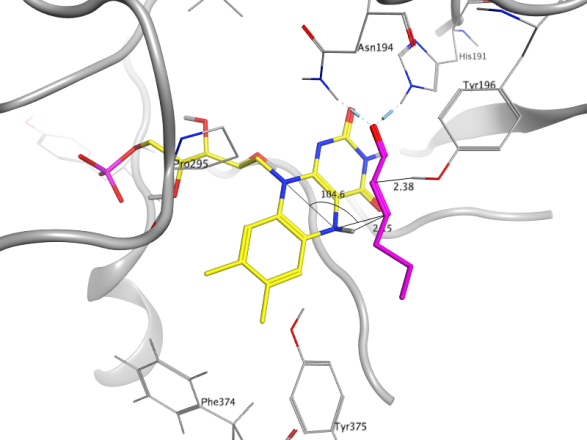 | 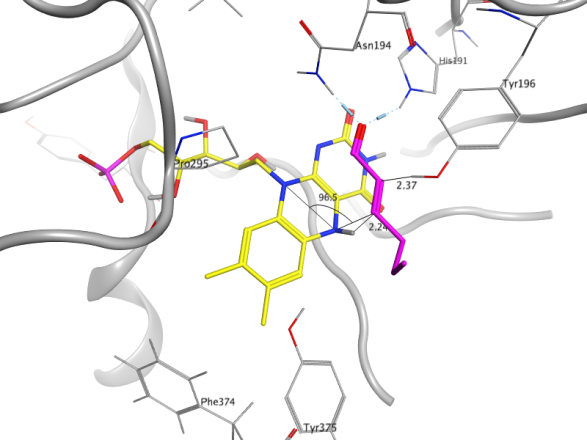 |
| Pose entry 4  Accepted | Pose entry 6  Accepted |
