# Supplementary material for: In silico and in vitro studies of the reduction of unsaturated α,β bonds of trans-2-hexenedioic acid and 6-amino-trans-2-hexenoic acid – Important steps towards biobased production of adipic acid
Source: PLoS One. 2018 Feb 23;13(2):e0193503. doi: 10.1371/journal.pone.0193503 (PMC5825115; doi:10.1371/journal.pone.0193503)
Supplement: S1 Supporting information — (DOCX) [file pone.0193503.s005.docx]

**S1 Supporting information.**

**Materials and methods.**

**Heat treatment of 6-amino-*trans*-2-hexenoic acid.**

To investigate spontaneous ring-closure, samples of 6-amino-*trans*-2-hexenoic acid heat treated (30 °C, 4h) were analysed on an ion chromatography. After heat treatment of 100 µM 6-amino-*trans*-2-hexenoic acid, an unknown peak emerged at RT ca 6 minutes (Figure S 1 A) whereas the standard was known to elute at RT ca 9.5 minutes. When 6-amino-*trans*-2-hexenoic acid was boiled the peak shifted completely from RT ca 9.5 minutes to RT ca 6 minutes (Figure S 1 B) indicating that 6-amino-*trans*-2-hexenoic acid is formed to something else when heated.

# **Synthesis of *trans*-2-hexenedioic acid.**

Acrylic acid (0.94 ml, 14.4 mmol) and 4-pentenoic acid (0.7ml, 6.86mmol) was dissolved in Dichloromethane, DMC, (18ml) To the mixture was **(1,3-Bis(2,4,6-trimethylphenyl)-2-imidazolidinylidene)dichloro(phenylmethylene)(tricyclohexylphosphine)ruthenium (**Grubb’s catalyst 2^nd^ generation) (90mg, 0.14mmol) added and the mixture was refluxed for 4 hours. During the reaction the product precipitated out from the reaction mixture. After cooling to room temperature the product was filtered off and was washed with heptane.

Yield *trans*-2-hexenedioic acid 225mg, 22.5%.

2.38, td, J=8, 2 Hz, 2H, 2.41, t, J=8 Hz, 5,76, dt, J=16, 2 Hz, 1H, 6.87, dt, J=16, 8 Hz, 1H

13C 26.6, 31.7, 120.9, 149.5, 170.4, 177.3
