# Supplementary figures and images for: In silico and in vitro studies of the reduction of unsaturated α,β bonds of trans-2-hexenedioic acid and 6-amino-trans-2-hexenoic acid – Important steps towards biobased production of adipic acid
Source: PLoS One. 2018 Feb 23;13(2):e0193503. doi: 10.1371/journal.pone.0193503 (PMC5825115; doi:10.1371/journal.pone.0193503)

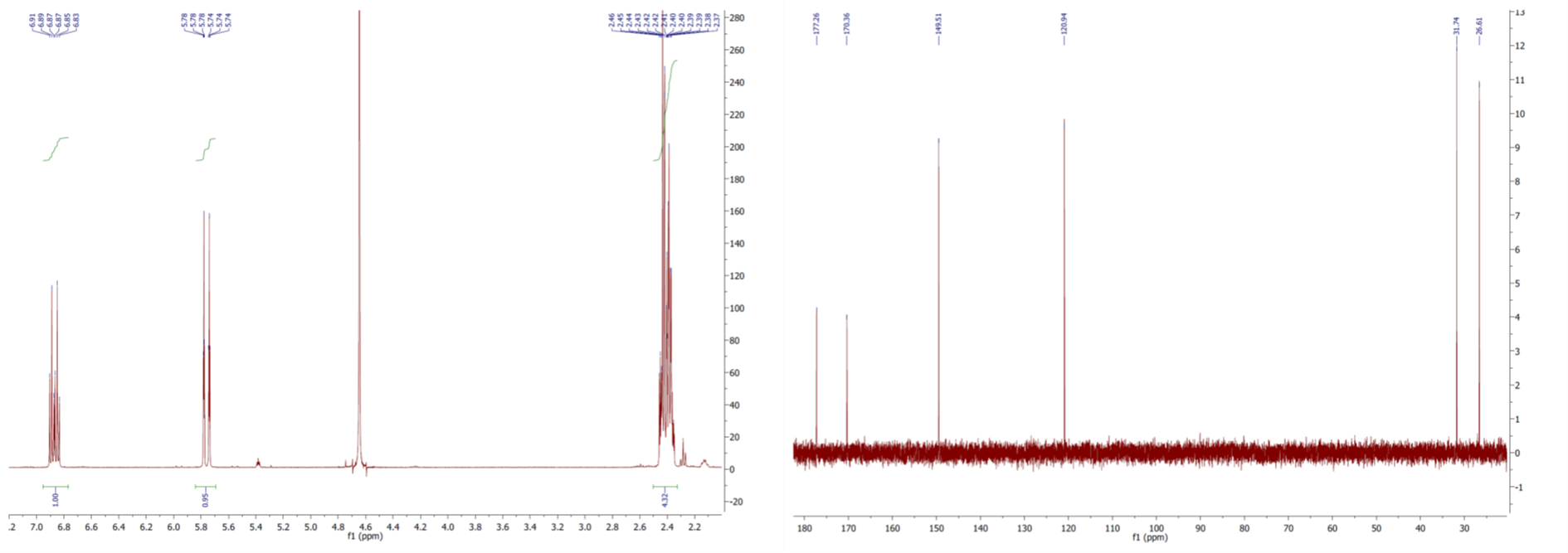

Supplement: S1 Fig — (TIF) [file pone.0193503.s006.tif]

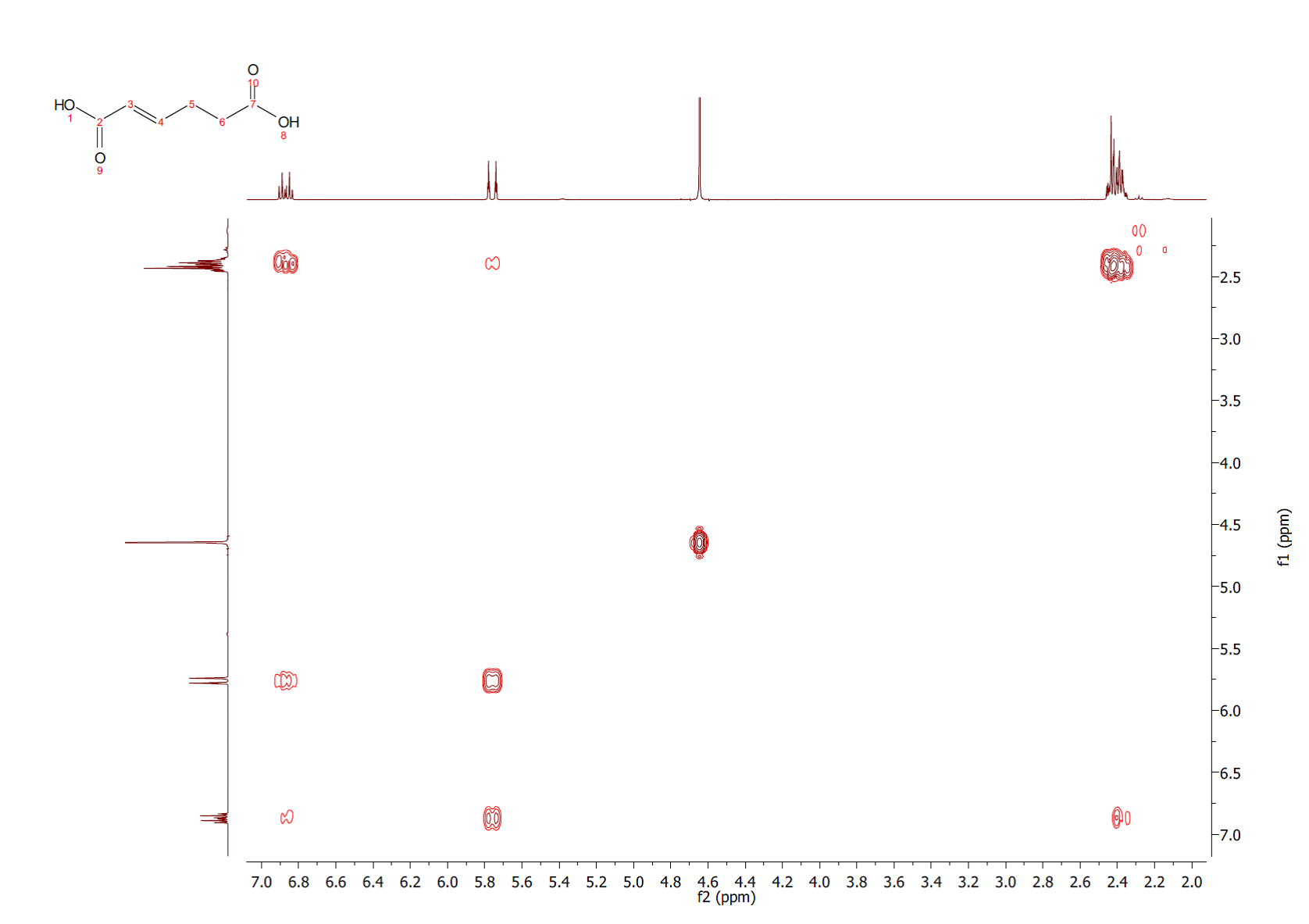

Supplement: S2 Fig — (TIF) [file pone.0193503.s007.tif]

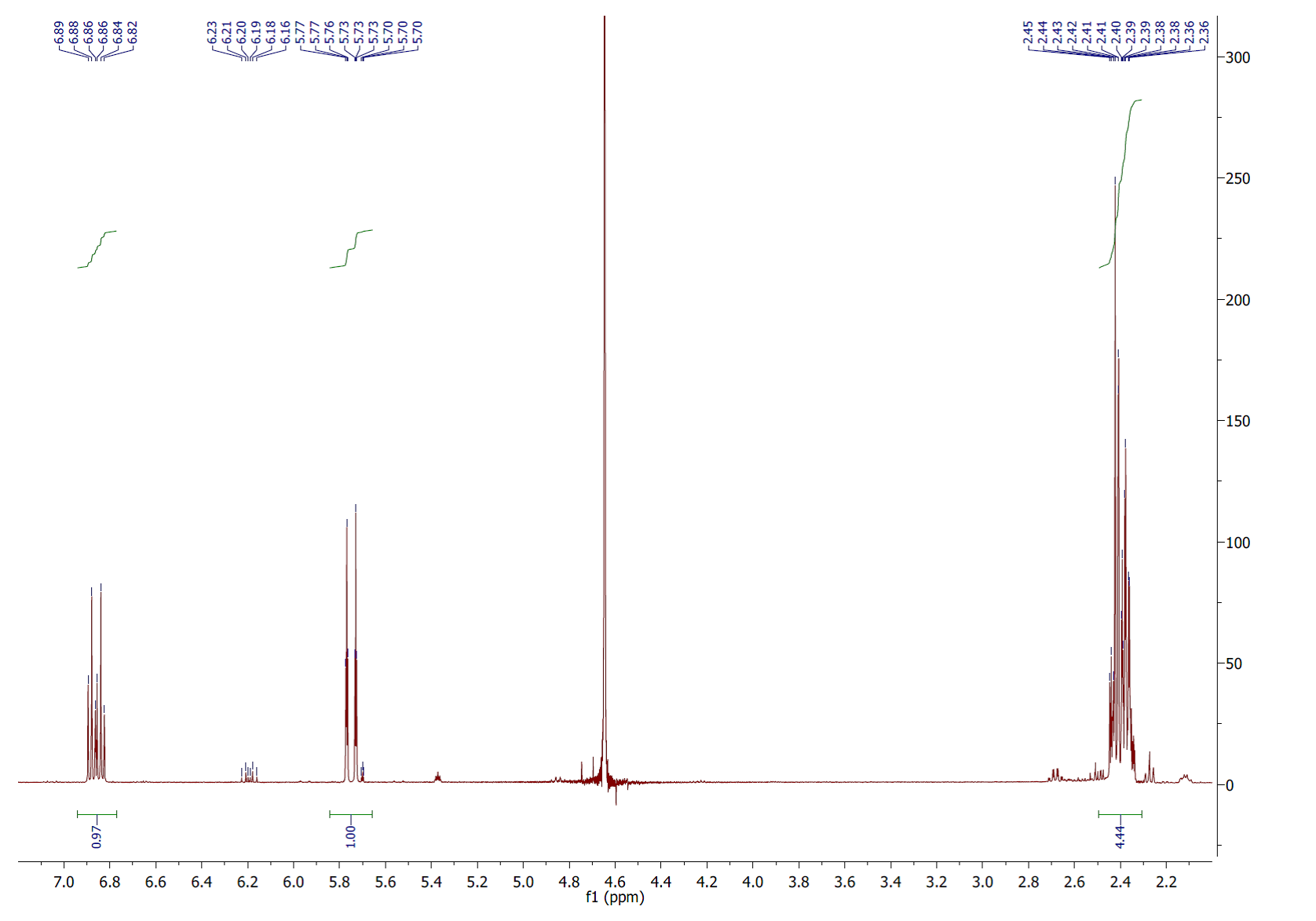

Supplement: S3 Fig — trans-2-Hexenedioic acid was heated at 90°C for 6h. No intramolecular reaction was detected, only a small, less than 5%, isomerization from trans to cis was observed by new signals for the cis-alkene at 6.19, td, J = 12, 8 Hz, 5.71 dt, J = 12, 2 Hz. (TIF) [file pone.0193503.s008.tif]

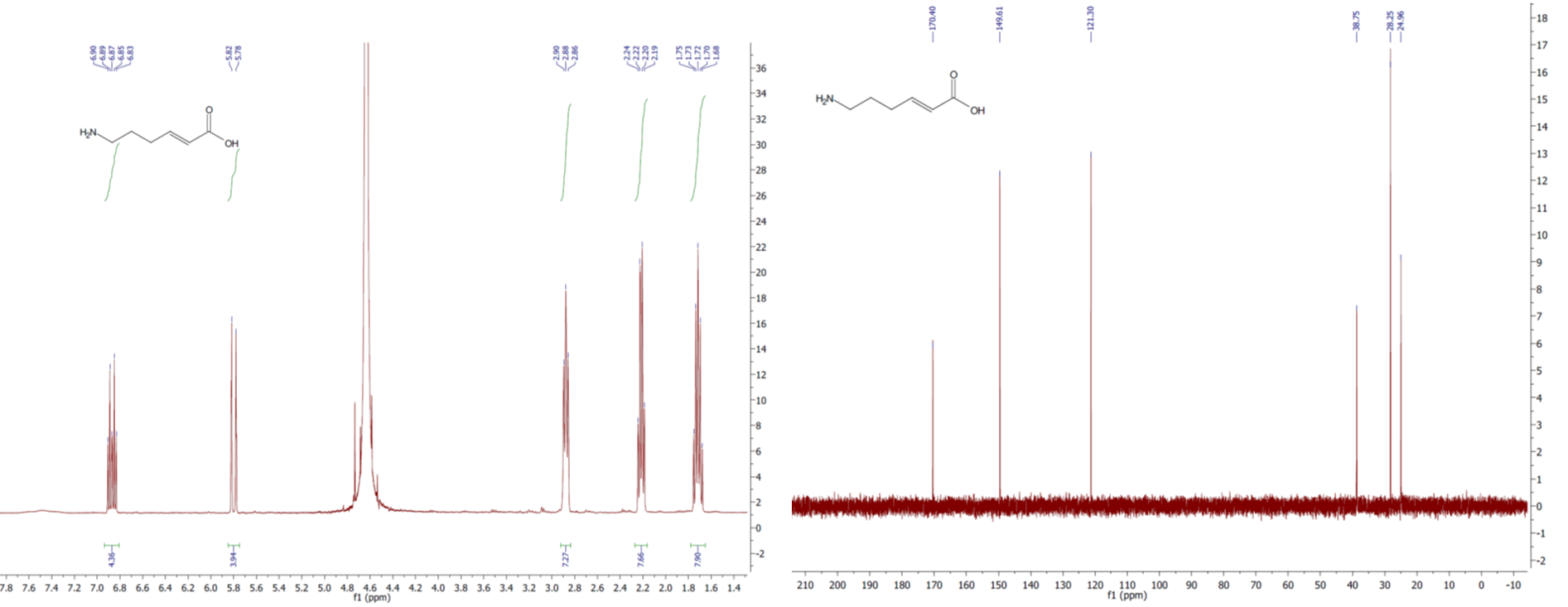

Supplement: S4 Fig — (TIF) [file pone.0193503.s009.tif]

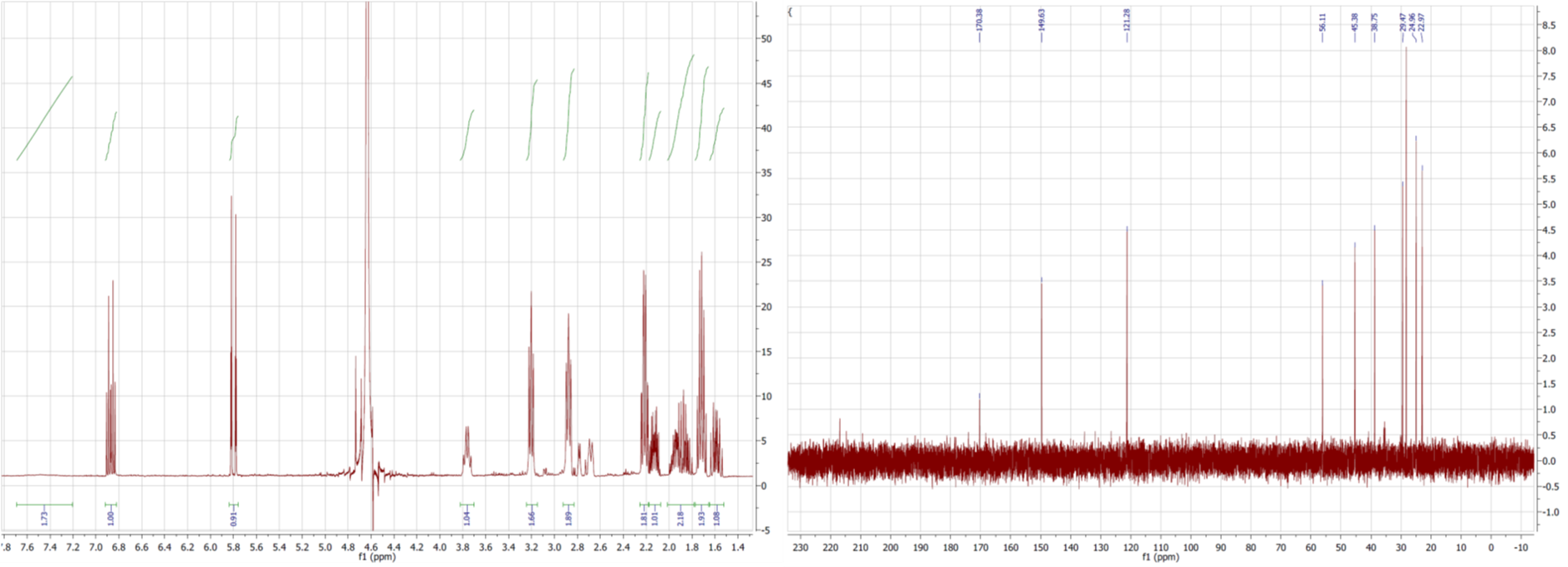

Supplement: S5 Fig — (TIF) [file pone.0193503.s010.tif]

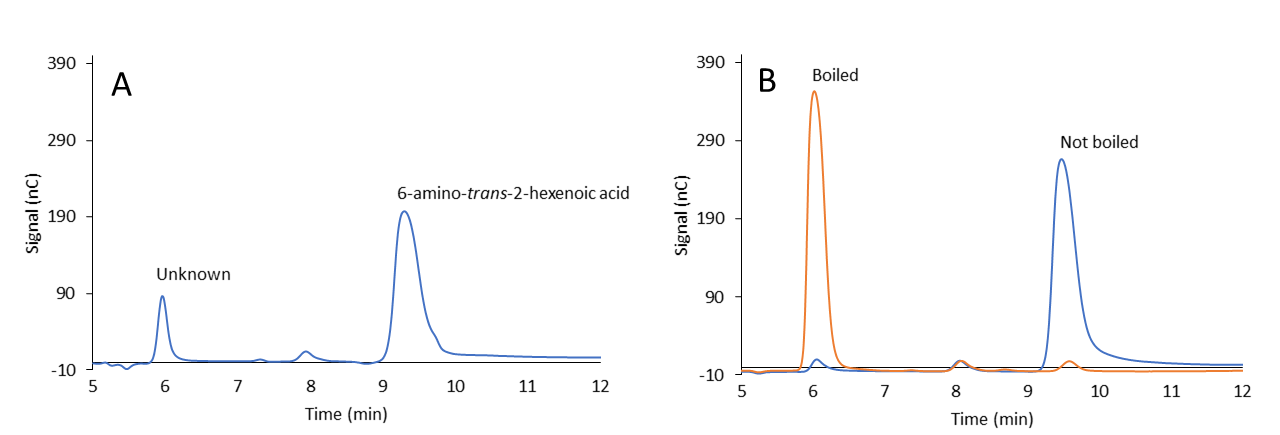

Supplement: S6 Fig — A) 100 μM 6-amino-trans-2-hexenoic acid, treated at 30°C for 4 hours. B) 100 μM 6-amino-trans-2-hexenoic acid 100 μM (not boiled) (blue) and 6-amino-trans-2-hexenoic acid 100 μM boiled (orange). (TIF) [file pone.0193503.s011.tif]

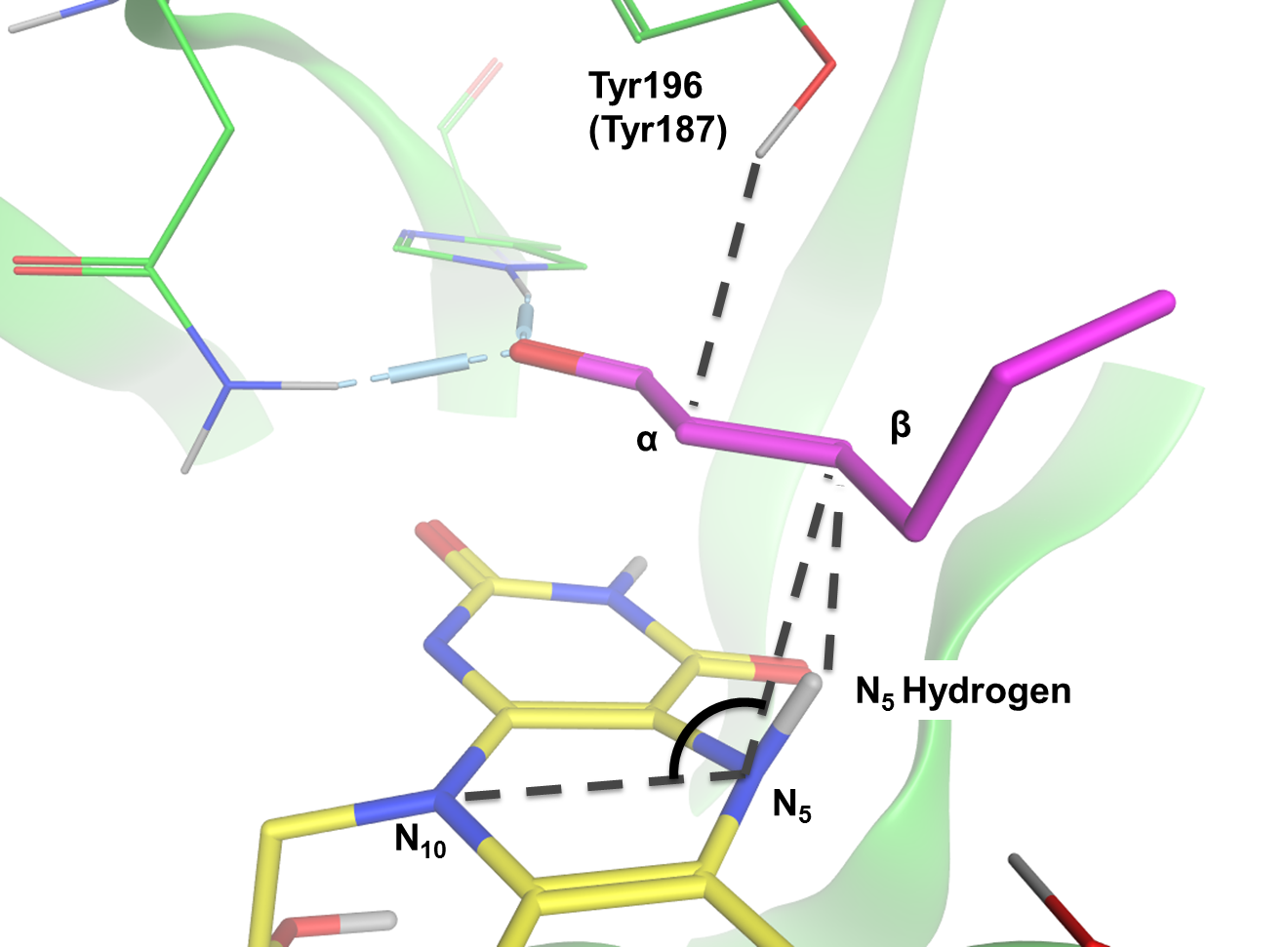

Supplement: S7 Fig — Distance between hydroxyl hydrogen of Tyr196 of Oye1 (Tyr187 for NemA) and alpha carbon is measured. Distance between N5 hydrogen and the beta carbon is also measured (threshold 3.96 Å). The angle is defined by N10-N5-beta carbon. The accepted range for this angle is 86.0° - 111.8°. (TIF) [file pone.0193503.s012.tif]

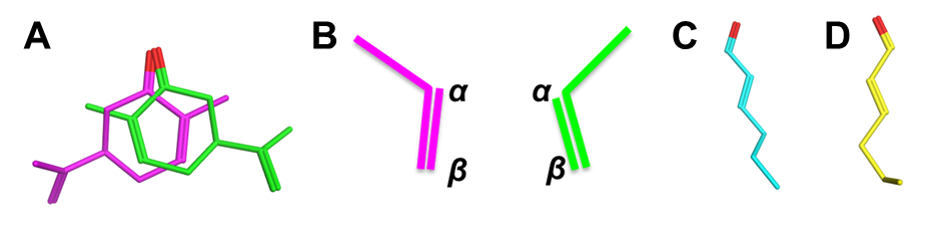

Supplement: S8 Fig — (A) Illustration of normal binding mode (magenta) and flipped binding mode (green). Coordinates are extracted from previously described reports (PDB = 4GWE and 4GE8) [51]. (B) Determination of binding mode according to the C1 position respect to α and β carbons. (C) Exemplary normal binding result for trans-2-hexenal (cyan). (D) Exemplary flipped binding mode for trans-2-hexenal (yellow). (TIF) [file pone.0193503.s013.tif]

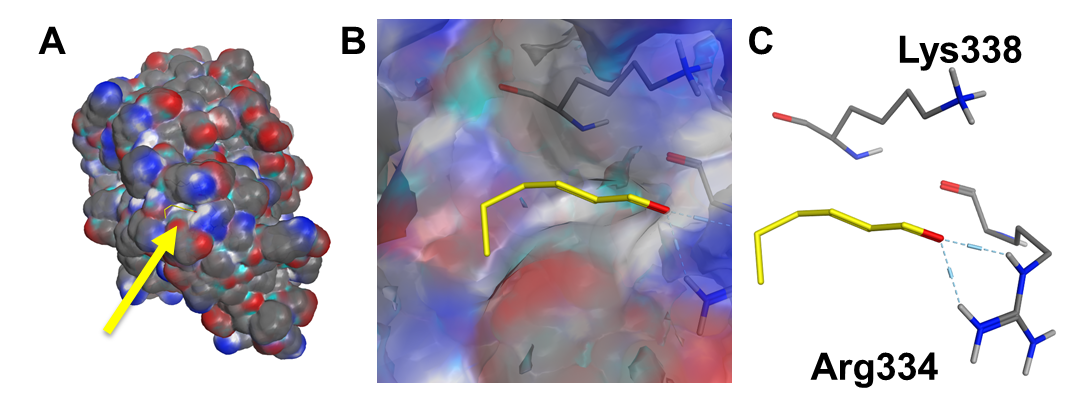

Supplement: S9 Fig — Potential interaction with positive patch on enzyme surface and substrate is shown. A) Overall electrostatic view of 1OYB with bound trans-2-hexenal on the 1OYB surface. Yellow arrow indicates the relative position of the electrostatic interaction. B) Zoomed-in view of the interaction of bound trans-2-hexenal (yellow) and the 1OYB enzyme. C) Hydrogen bonding of Lys338 and Arg334 to carbonyl group of trans-2-hexenal. (TIF) [file pone.0193503.s014.tif]

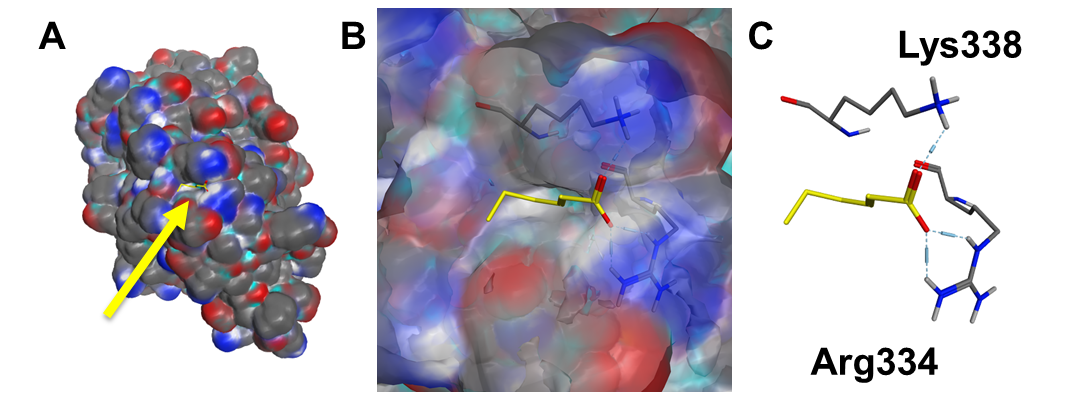

Supplement: S10 Fig — Potential interaction with positive patch on enzyme surface and substrate is shown. A) Overall electrostatic view of 1OYB with bound trans-2-hexenoate on the 1OYB surface. Yellow arrow indicates the relative position of the electrostatic interaction. B) Zoomed-in view of the interaction of bound trans-2-hexenoate (yellow) and the 1OYB enzyme. C) Hydrogen bonding of Lys338 and Arg334 to carboxylate group of trans-2-hexenoate. (TIF) [file pone.0193503.s015.tif]

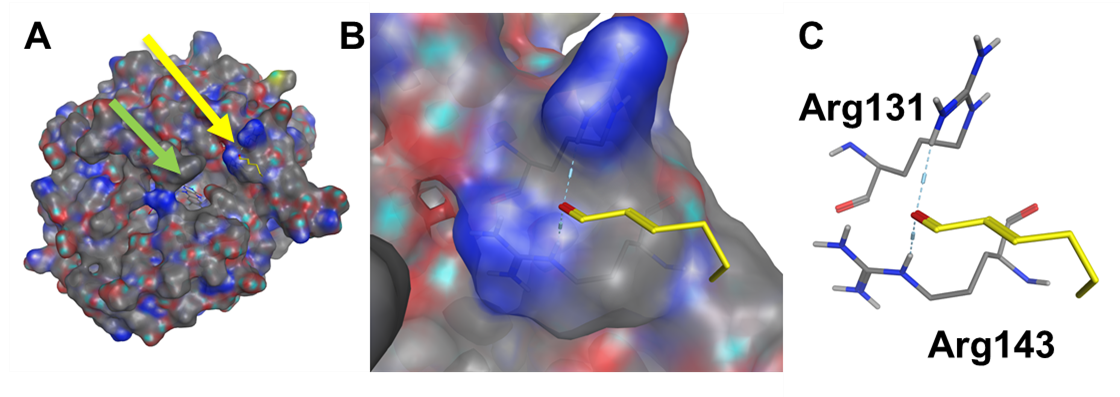

Supplement: S11 Fig — Potential interaction with positive patch on enzyme surface and substrate is shown. A) Overall electrostatic view of NemA with bound trans-2-hexenal on the NemA surface. Yellow arrow indicates the relative position of the electrostatic interaction. Green arrow indicates the catalytic pocket. B) Zoomed-in view of the interaction of bound trans-2-hexenal (yellow) and the NemA enzyme. C) Hydrogen bonding of Arg131 and Arg143 to carbonyl group of trans-2-hexenal. (TIF) [file pone.0193503.s016.tif]

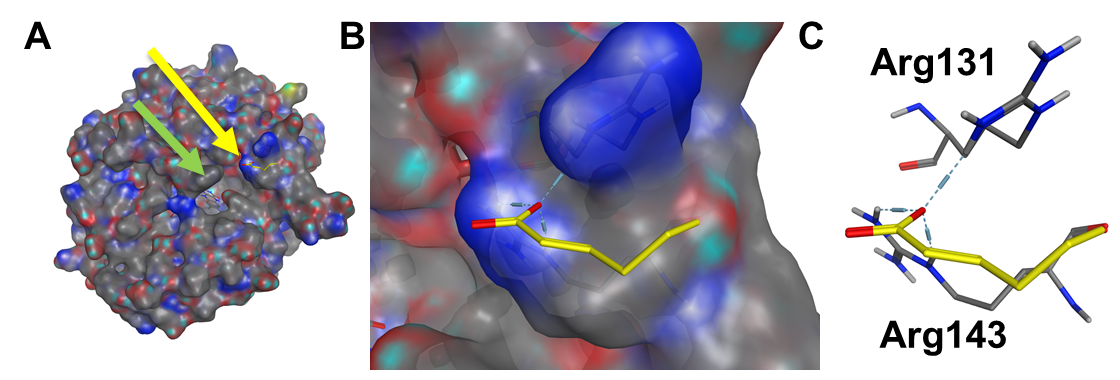

Supplement: S12 Fig — Potential interaction with positive patch on enzyme surface and substrate is shown. A) Overall electrostatic view of NemA with bound trans-2-hexenoate on the NemA surface. Yellow arrow indicates the relative position of the electrostatic interaction. Green arrow indicates the catalytic pocket. B) Zoomed-in view of the interaction of bound trans-2-hexenoate (yellow) and the NemA enzyme. C) Hydrogen bonding of Arg131 and Arg143 to carboxylate group of trans-2-hexenoate. (TIF) [file pone.0193503.s017.tif]

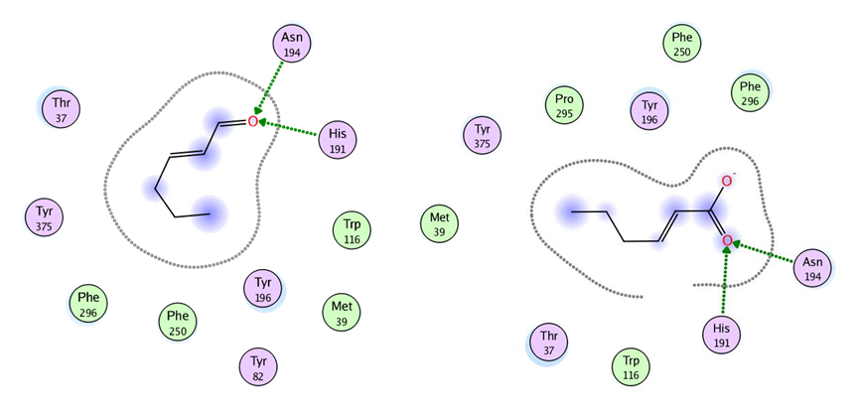

Supplement: S13 Fig — Green arrows indicate hydrogen bonding between the enzyme and substrate. (TIF) [file pone.0193503.s018.tif]
